# Reducing the Impacts of Biofouling in RO Membrane Systems through In Situ Low Fluence Irradiation Employing UVC-LEDs

**DOI:** 10.3390/membranes10120415

**Published:** 2020-12-11

**Authors:** Philipp Sperle, Christian Wurzbacher, Jörg E. Drewes, Bertram Skibinski

**Affiliations:** Chair of Urban Water Systems Engineering, Department of Civil, Geo and Environmental Engineering, Technical University of Munich, Am Coulombwall 3, 85748 Garching, Germany; philipp.sperle@tum.de (P.S.); christian@wurzbacher.cc (C.W.)

**Keywords:** biofouling, reverse osmosis membrane, ultra violet irradiation, LED, microbial diversity

## Abstract

Biofouling is a major concern for numerous reverse osmosis membrane systems. UV pretreatment of the feed stream showed promising results but is still not an established technology as it does not maintain a residual effect. By conducting accelerated biofouling experiments in this study, it was investigated whether low fluence UV in situ treatment of the feed using UVC light-emitting diodes (UVC-LEDs) has a lasting effect on the biofilm. The application of UVC-LEDs for biofouling control is a novel hybrid technology that has not been investigated, yet. It could be shown that a low fluence of 2 mJ∙cm^−2^ delays biofilm formation by more than 15% in lab-scale experiments. In addition, biofilms at the same feed channel pressure drop exhibited a more than 40% reduced hydraulic resistance. The delay is probably linked to the inactivation of cells in the feed stream, modified adsorption properties or an induced cell cycle arrest. The altered hydraulic resistance might be caused by a change in the microbial community, as well as reduced adenosine triphosphate levels per cells, possibly impacting quorum sensing and extracellular polymeric substances production. Due to the observed biofilm attributes, low fluence UV-LED in situ treatment of the feed stream seems to be a promising technology for biofouling control.

## 1. Introduction

Many regions worldwide suffer from water scarcity [1,2]. Especially if no conventional water resources are available, water reuse [3], brackish groundwater or seawater desalination are viable approaches to provide alternative water supplies [4,5,6]. In these scenarios, pressure-driven membrane filtration, including nanofiltration (NF) and reverse osmosis (RO), may act as a key process while employing advanced water treatment [5,7]. Membranes are an attractive technology as they can reject both organic and inorganic contaminants as well as remove pathogens [8]. However, fouling, a loss of membrane performance over time, is a significant drawback [9,10].

For many NF and RO applications, the presence of microorganisms and nutrients in the feed stream, especially biofouling, is a major concern [11,12,13,14,15]. Biofouling is defined as an undesired biofilm formation leading to operational challenges, such as a higher pressure drop, a reduction in membrane flux, an increased salt passage and/or a reduced membrane lifetime [15,16,17]. The biofilm is built up in three phases: (1) adhesion and attachment of microorganisms, (2) their growth and (3) a stationary phase [15] (p. 13). Amongst others, the adhesion phase is influenced by the microorganism species, their population density and physiological responses, as well as the concentration and composition of dissolved organic matter in the feed water [18,19].

The biofilm itself consists of microorganisms (usually ≤10% of dry mass) and a matrix known as extracellular polymeric substances (EPS) [20]. Biofilms forming on membrane surfaces comprise a broad microbial community, of which the composition is influenced by the influent water matrix, degree of pretreatment, temperature, and membrane type employed [21]. Furthermore, the biofilm community can change with age [21,22]. Commonly found microorganisms on high pressure membranes include, among others, Alphaproteobacteria (e.g., Order/Family/Genus of Rhizobiales/Bradyrhizobiaceae/*Rhodopseudomonas* or Sphingomonadales/Sphingomonadaceae/*Sphingomonas*) [21,23,24,25,26,27,28], *Betaproteobacteria* (e.g., Burkholderiales/Comamonadaceae/*Hydrogenophaga*, *Polaromonas* and *Acidovorax)* [21,28] and Gammaproteobacteria (Pseudomonadales/Pseudomonadaceae/*Pseudomonas* or Xanthomonadales/Xanthomonadaceae/*Pseudoxanthomonas*) [11,21,23,24,28,29,30,31,32,33,34,35,36,37]. Furthermore, de Vries et al. [28] revealed that the community in the feed water and the corresponding biofilm differs. Microorganisms present in the fouling layer are usually only present in a small relative abundance in the feed. The composition of EPS, representing the ‘glue’ of the three dimensional architecture of the biofilms, varies depending on microorganisms themselves, shear forces, temperature, and nutrients [20]. Aside from keeping the microbial cells together, EPS has several functions, allowing intense interactions, cell-cell communication (quorum sensing), the formation of synergistic micro-consortia, adhesion and retaining extracellular enzymes [20]. By keeping lysed cells and DNA in the matrix, it also enables horizontal gene transfer [20]. The EPS biopolymers include proteins, polysaccharides, nucleic acids, lipids and other organic matter fractions such as humic substances [20,38]. The EPS matrix can vary in its concentration, cohesion, charge, sorption capacity and architecture [20]. The morphology can vary in its porosity, as well as being smooth, flat, rough, fluffy or filamentous. Depending on hydrodynamic conditions, nutrient supply, bacterial motility, intercellular communication/quorum sensing or exopolysaccharides and proteins, the biofilm attributes can change [20]. Within quorum sensing, *N*-acyl homoserine lactones (AHLs) molecules are commonly found [39]. Dreszer et al. rather linked EPS to the hydraulic resistance of biofilms than to the cells alone [40]. Herzberg and Elimelech [41] further revealed that (dead) cells are responsible for an increased salt passage by hindering the back diffusion of salts, leading to a higher osmotic pressure difference on the membrane surface, whereas EPS is mainly responsible for an increased hydraulic resistance.

Currently, there are different approaches to mitigate the unwanted effects of biofouling. These include:

1. Adaption of the equipment, design and operation, such as suitable pretreatment (e.g., ultrafiltration), optimizing hydrodynamic conditions, membrane surface modifications or feed flow reversal,
2. Limitation of the biomass growth conditions by limiting essential resources, such as carbon or phosphorus,
3. Application of cleaning agents, such as sodium hydroxide, monochloramine or other biocidal chemicals [15] (p. 291), [18,42].

Each of the biofouling mitigation strategies has its own strengths and weaknesses. Whereas adaption of the equipment, design and operation, as well as the limitation of biomass growth conditions, can delay and reduce the negative impacts of biofouling, applying cleaning agents has its advantage in removing already-established biofilms. Nevertheless, for cleanings it is important to remove the dead biomass, as this could otherwise lead to fast regrowth, and depending on the used chemical, the membrane might get deteriorated [17,18,31,43,44]. According to Vrouwenvelder et al. [43], biofouling shall ideally be controlled through a combination of single approaches but without the need of cleaning agents. Unfortunately, this might not be possible at the current state. Thus, even though there exist a variety of strategies for biofouling control, through performance losses, higher energy demand, downtimes in production, cleaning chemicals and their disposal, reduced membrane lifespan and other operational problems, biofouling is still associated with high costs [14]. Hence, there still is a great need for alternative, efficient biofouling mitigation strategies.

UV irradiation using mercury vapor lamps is a well-known approach for the inactivation of microorganisms. The inactivation is mainly caused by damage to the DNA through the formation of dimers [45,46,47,48]. Other secondary effects caused by UV irradiation are direct and indirect damage to the cells’ biomolecules by producing reactive oxygen species (ROS) or exiting photosensitizer molecules within or outside the cell [49]. Pullertis et al. [50] investigated the impact of UV disinfection on the microbial community of drinking water and its development during subsequent storage for six days at 7 °C. According to their findings, the community after storage changed significantly. The guanine and cytosine (GC) content of bacterial genomes could be an indicator of UV resistance [50,51,52]. Furthermore, gram-positive and spore-forming organisms could be more UV resistant [50,53,54,55]. Aside from inactivation, in experiments using solar UV and UV-A disinfection, it was reported that adenosine triphosphate (ATP) levels per cell, efflux pump activity, membrane potential and glucose uptake were reduced [56]. Matallana-Surget and Wattiez [57] reviewed that there are several different responses from microorganisms subjected to UV irradiation (DNA damage), including cell cycle arrest, repair pathways, stress responses, damage tolerance and cell death. In general, (solar) UV radiation can lead to protein damage due to oxidative stress, including amino acid modifications, carbonyl group formation and protein-protein cross-links [57].

Taking into account the various effects of UV irradiations on cells, UV disinfection of the feed stream could be a viable strategy to control biofouling. In particular, RO feed water is usually pretreated to a great extent, and hence, it commonly exhibits high UV transmissions, making it ideal for UV disinfection. Studies investigating the potential of UV-disinfection pretreatment used low or medium pressure lamps, with fluences of 20–400 mJ∙cm^−2^ [58,59,60,61]. In several studies, it could be demonstrated that the UV-disinfection is delaying the build-up of the biofilm in NF or RO membrane systems using river or groundwater as feed in a full-scale application [58,59,60]. Furthermore, biofilms grown during a defined period of time exhibited a lower dry weight and cell number as well as a reduced ATP, protein and polysaccharides content [60]. In addition, Harif et al. [58] recognized a lower specific EPS production per biovolume and an altered microbial community composition. In particular, Harif et al. [58] reported a less diverse community. Especially the Alphaproteobacteria genus of *Parvularcula* as well as Betaproteobacteria and Chloroflexi exhibited an increased abundance in the UV treated biofilm. Alphaproteobacteria in general were not affected. On the contrary, Nitrospirae and Bacteroidetes were reduced. The reason for a positive effect for biofouling control could be attributed to a reduced viable cell count in the feed stream, as well as a change in the adhesion properties [58,60,62], with the exception of Lakretz et al. [63], who observed a reduced membrane flux with UV pretreatment.

So far, UV irradiation as a pretreatment strategy for high-pressure membrane applications has not been examined in great depth, probably because UV disinfection of the feed stream has no residual effect on the downstream biofilm [42,64]. Yet, it is not clear if the observed changes in the biofilm, such as lower EPS production per biovolume [58], are only linked to a delay of the biofilm formation, as experiments were frequently terminated after a defined period of time [58,59,60] rather than a function of biofouling development.

Moreover, apart from low and medium pressure lamps, recent developments in the semiconductor sector led to the emerged application of UVC light-emitting diodes (UVC-LEDs) [65]. These LEDs offer the opportunity to explore economically new designs and operations for in situ UV treatments as they are compact and robust, potentially more energy efficient with an increased life span, as well as they do not use or potentially release toxic mercury [65,66]. The combination of higher lifetime and energy efficiency with the possibility of new reactor designs are of great interest as this could enable an in situ integration of the LEDs into the pressure vessel of RO systems.

**Figure 1:**
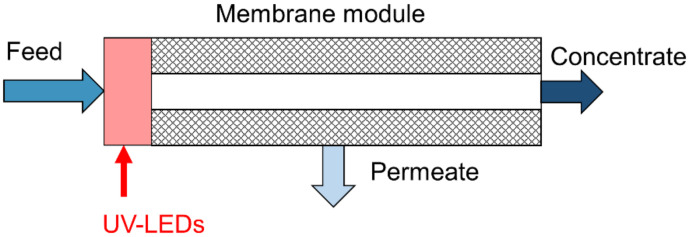
Schematic application of UV light-emitting diodes (UV-LEDs) for in situ biofouling control.

This study aims to elucidate the potential of UVC irradiation using the recently developed UV-LEDs as an in situ pretreatment strategy for biofouling control in RO or NF systems (Figure 1). In contrary to UV studies carried out previously, we will test if low fluences are sufficient to not only delay the biofilm formation but further lead to a reduced hydraulic resistance of the biofilm while approaching a severe biofouling state. To examine the mechanisms behind a change in hydraulic resistance, the biofilm formed will be analyzed for changes in ATP level, EPS composition and microbial diversity. We hypothesize that in addition to a delay of the biofilm formation by inactivating microorganisms in the feed stream, the structure of the biofilm is changed with lasting impacts. If the effect of UV treatment is enduring, it would present an environmentally friendly biofouling control strategy, saving chemicals, energy and costs.

## 2. Materials and Methods

### 2.1. UV Reactor and Laboratory Skid for Biofouling Experiments

The flow-through UVC-LED reactor consisted of an LED located directly over a silica glass pipe (7 mm diameter, 50 mm length), covered by an ABS housing. The LED type LEUVA66H70HF00 manufactured by LG Innotek (Seoul, Korea) has its peak wavelength in a range of 270–285 nm (typically 278 nm) with spectrum half width of 10 nm, a view angle of 110° and an optical power of 110 mW [67]. An AMPYR LED30W controller (Lighting & Electronics Jena, Jena, Germany) was used to set the driving current, automatically adapting the needed voltage.

**Figure 2:**
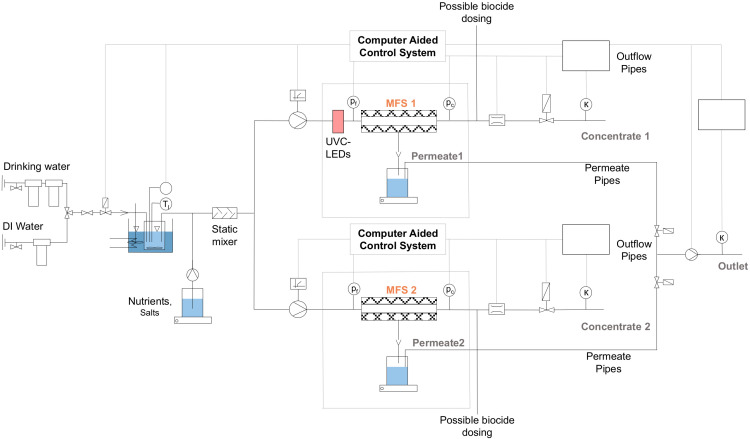
Schematic drawing of the laboratory scale skid for biofouling experiments (further details of the operation are given in the Supplementary Materials [68]). MFS: membrane fouling simulator; DI: deionized

To investigate the potential of in situ UV pretreatment for biofouling control, accelerated biofouling experiments employing membrane fouling simulators (MFS) [15] were performed. A low-pressure RO membrane LOW1 (Oltremare, Fano (PU), Italy) was used, of which an active membrane area of 2 × 12 cm was employed in an MFS (Figure 2). The feed spacer had a height of 20 mil in diamond shape with a porosity of 0.775 and a contact angle of 45°. The UVC-LED reactor was placed in immediate vicinity (<10 cm) of the MFS, thus mimicking an in situ integration in the pressure vessel of an up-scaled system. A “UV-reactor dummy,” simply a silica glass pipe with the same dimensions as used in the UV reactor, was attached to the reference train to ensure the same hydraulic retention times in the system. A detailed description of the skid is presented in the Supplementary Materials Figure S1 and Table S2 [68].

### 2.2. Accelerated Biofouling Experiments

In order to quantify a delay in the biofilm build-up, a certain termination criterion needs to be defined. As neither the cell number within the biofilm nor the total biomass could be monitored online with the available methods, it was decided to take the feed channel pressure drop (FCPD) as a reference value. In previous studies, it was observed that with increasing biomass the FCPD rises [15,69,70]. Moreover, membrane manufacturers advised taking action against biofouling if an FCPD increase exceeding 15% is observed [15] (p. 22). Even though it might not be possible to link the FCPD directly to the biofilm volume, FCPD is a reasonable criterion to quantify the delay of biofilm formation up to a certain degree of biofouling. In preliminary experiments in the 12 cm long MFS, the plateau phase of the FCPD started at values exceeding 1.5 bar. An arbitrary value of 0.8 bar (67 mbar∙cm^−1^) was chosen as the terminating criterion to be still in the exponential FCPD increase and to have a sufficient biofilm volume to monitor changes in the biofilm composition.

For the accelerated biofouling experiments, local tap water (groundwater, treated by aeration and sand filtration) under the addition of easily degradable nutrients 1000:200:100 μg∙L^−1^ of C:N:P [40] was used as feed. Sodium acetate served as a C source, sodium nitrate as an N source and sodium dihydrogen phosphate dihydrate as a P source (all supplied by Merck, Darmstadt, Germany) supplemented to the tap water. The workflow for the biofouling experiments consisted of four basic steps (see Supplementary Materials for a detailed description [68]):

1. Cleaning and sterilization: Membranes were stored at 4 °C in 1% NaHSO_3_ (Acros Organics, Geel, Belgium) until used [71]. The cleaning of the system included flushing with 0.1% NaOH (Merck, Darmstadt, Germany) to remove organic matter [71]. Sterilization was done by autoclaving at 121 °C for 20 min or soaking in 0.25% H_2_O_2_ solution (Merck, Darmstadt, Germany), analogous to [71,72,73,74,75].
2. Reassembling of the skid: After cleaning and sterilization, the skid was reassembled under sterile conditions.
3. Compaction: Before starting each biofouling experiment, a 16-h compaction, using an NaCl solution (Appli Chem, Darmstadt, Germany), was performed and the flux was set to 20 L∙m^−2^∙h^−1^ (LMH). Depending on the initial membrane permeability, the feed pressure varied between 3.5 and 6 bar. The crossflow through the MFS is maintained at 4.25 L∙h^−1^ resulting in an empty channel velocity of 0.116 m∙s^−1^.
4. Accelerated biofouling phase: Biofouling experiments were performed by using tap water as feed with nutrient dosing to reach a target concentration of 1000:200:100 μg∙L^−1^ of C:N:P [40]. Temperature was maintained at 15 °C and feed pressure (set in compaction) was kept constant. When an FCPD of 0.8 bar was reached, the experiments were terminated.

### 2.3. Biofilm Extraction and Analysis

After terminating an experiment, the biofilm was extracted under sterile conditions and analyzed for the parameters shown in Table 1. Biofilm was extracted by cutting the membrane and spacer in a falcon tube containing 30 mL of 0.1 M NaCl and vortexing for 1 h similar as described by Matar et al. [76]. EPS was extracted by ultrasonic treatment [77] and centrifugation [76]. A detailed description is provided in the Supplementary Materials [68].

### 2.4. Analytical Methods

The methods used for analysis are the following:

- Total organic carbon (TOC) was analyzed as a non-purgeable organic carbon using the Vario TOC cube analyzer (Elementar Analysensyteme, Langenselbold, Germany) [79]. Biofilm samples were diluted 1:50 prior to analysis.
- ATP was measured using the BacTiter-Glow assay (Promega, Walldorf, Germany). The manufacturer’s guideline [80] was followed using flat white 96 multiwell plates (Thermo Scientific, Langenselbold, Germany) and the Tecan Infinite M Plex reader (Männedorf, Switzerland). ATP standards were prepared using adenosine 5′-triphosphat disodium salt (Sigma-Aldrich Chemie, Taufkirchen, Germany). As inner filter effects were observed during ATP analysis, each sample was diluted 1:5, 1:10 and 1:20. The luminescence signal was interpolated to an undiluted state and converted to ATP concentrations with the standard curve.
- Total direct cell counts (TDC) was performed following the procedure of Boulos et al. [81] using the LIVE/DEAD BacLight kit (Thermo Scientific, Langenselbold, Germany). For filtration, the 0.22 µm black polycarbonate filter manufactured by Piper Filter (Bad Zwischenahn, Germany) were utilized. With this kit, it is possible to differentiate cells with intact and damaged cell membrane [81]. Images were taken using the Axioplan 2 imaging employing the Axiocam 503 color camera (Zeiss, Oberkochen, Germany). Cell counting was performed using the Matlab Version (R2018b, Mathworks, Natick, MA, USA) of the CellC software v. 1.2 [79].
- Protein analysis was done using the modified Lowry protein assay kit (Thermo Scientific, Langenselbold, Germany). Transparent 96 well flat transparent microplates (Greiner Bio-One, Frickenhausen, Germany) were used with the Infinite M Plex reader (Tecan, Männedorf, Switzerland). The protein standard curve was prepared using bovine serum albumin (Thermo Scientific, Waltham, MA, USA).
- Polysaccharides were quantified using the method described by Masuko et al. [82]. Concentrated sulfuric acid was purchased from Merck (Darmstadt, Germany) and phenol from Sigma Aldrich (St. Louis, MO, USA). Standards were prepared using d-(+)-glucose (Alfa Aesar by Thermo Fisher Scientific, Kandel, Germany). The same multiwell plates and reader as for proteins were utilized.
- Fluorescence spectroscopy was performed using the Aqualog (HORIBA Jobin Yvon, Bensheim, Germany). Two different kinds of samples were analyzed: once the biofilm sample filtered by 0.45 µm (VWR, Radnor, PA, USA) and the EPS sample unfiltered. Both samples were diluted 1:20 to reduce inner filter effects. Instrument settings are summarized in the Supplementary Materials [68]. QS high precision cell made of quartz SUPRASIL by Hellma (Müllheim, Germany) was utilized as cuvette.
- For 16S rRNA amplicon sequencing, 1.5 mL of sample was freeze dried, resuspended in 50 µL nuclease-free water (Promega, Walldorf, Germany) and DNA was extracted using the DNeasy PowerSoil kit (Qiagen, Hilden, Germany). The rRNA sequencing was performed by ZIEL—Institute for Food & Health (Freising, Germany) using the primers 341F/806R and a MiSeq Reagent Kit v3 on an Illumina MiSeq benchtop sequencer (Illumina, San Diego, CA, USA). Raw reads were uploaded to the European Nucleotide Archive (ENA) database (PRJEB41202 (ERP124942)).
- Anion and cation analyses were performed according to Standard Methods [83,84,85,86,87]. Feed water characteristics are presented in Supplementary Materials (Table S1) [68].

### 2.5. Summary of the Performed Biofouling Experiments

To verify the effects of in situ UVC treatment of the feed stream for biofouling control, in total eight biofouling experiments were performed. The first seven were performed with the maximum UVC-LED driving current of 360 mA, in the eighth experiment only 180 mA was used. The first experiment could not be stopped on time, so one biofilm analysis (except 16S rRNA sequencing) could not be performed. Experiment 3 had operational problems, thus only the biofilm analysis is available for that run. Experiment 7 was run only with intermittent permeate production (4 days with permeate production for membrane conditioning, then only about 2–3 h per day and finally the last hours during FCPD increase). This approach followed a recommendation by Vrouwenvelder et al. [15] (pp. 62–63) to clearly depict the effects of biofouling. They did not observe an influence of permeate production on biofouling, but it might reduce the impacts of other kinds of fouling. Still, this setting was only used once, as the aim of the study is to show the effects on fouling as close to real applications as possible. This resulted in six data sets for the membrane performance and biofilm attributes at a driving current of 360 mA. Table 2 summarizes the settings of these experiments.

### 2.6. Actinometry

Actinometry using KI/KIO_3_ was performed to measure the fluence delivered by the UVC-LED reactor to the water. The protocol was adapted from [88,89,90,91] and is shown in greater detail in the Supplementary Materials [68]. A similar but simpler, laboratory-scale skid was set up to perform actinometry experiments; details are given in the supplemental information Figure S3 and Table S3 [68]. The setup used consisted of a water bath, a pump, a flowmeter, a pressure sensor and a needle valve. To perform the actinometry experiments, a 0.6 M KI (Merck, Darmstadt, Germany), 0.1 M KIO_3_ (VWR, Darmstadt, Germany) and 0.01 M Na_2_B4O_7_ 10 H_2_O (Sigma-Aldrich, Taufkirchen, Germany) solution was prepared [89]. The pH of the finished solution was 9–9.2. The solution was adjusted to 23.5 °C in a temperature-controlled water bath, the temperature in the cabinet was set to 15 °C and UVC-LED reactor was turned on for at least 15 min to allow a warm-up period. Within an actinometry experiment, several flow steps were tested, ranging from approximately 1 to 6 L∙h^−1^ leading to hydraulic retention times in the reactor of 0.7 to 6 s. Samples and blank were taken in triplicates and the absorbance at 352 nm was determined using the DR6000 UV-Vis spectrophotometer (HACH, Düsseldorf, Germany).

### 2.7. Data Analysis

Outlier analysis for pressure data and calculated permeability (e.g., caused by an emptying of the beaker collecting the permeate) was done based on the interquartile range [92] (p. 14) of a 3 h interval. The detailed procedure is described in the Supplementary Materials [68].

Parallel factor (PARAFAC) modeling was performed using the Matlab based drEEM Toolbox V. 0.5.1 [93] available only at http://dreem.openfluor.org/. Components were identified using the OpenFuor database [94]. The PARAFAC modeling steps and the model itself are given in the supplement information [68]. The model was validated using variance explained, core consistency and split-half analysis [93,95,96].

16S rRNA amplicon sequencing data were processed using the R based DADA2 pipeline (V. 3.11) [97] and DECIPHER package 2.16.1 [98]. SILVA_SSU_r138_2019 was used as database, available online under: http://www2.decipher.codes/Downloads.html. In case a genus could not be assigned with the Silva database, the gene sequence was identified using BLAST [99,100] available online (https://blast.ncbi.nlm.nih.gov/Blast.cgi) against the INSDC databases [101]. For testing an overall change in the microbial community, microbial counts were rarefied [102,103] and compared in a distance-based redundancy analysis [104,105] by using the R package vegan 2.5–6 [106]. Dissimilarity was calculated using the Bray-Curtis distance. Furthermore, permutational multivariate analysis of variance using distance matrices (PERMANOVA) [107] using the vegan [106] package were applied to detect differences in the community. To test for differential abundance of single genera, generated count data were processed using the DESeq2 R package 1.28.1 [108], applying the “ashr” shrinkage estimator [109]. Corrections for false discovery rates within the calculations was performed according to Benjamini and Hochberg [110]. For Pearson correlation, data were transformed following the Rhea pipeline, applying a centered log ratio/scaling [111]. Hereby, only species present in 30% of the data and correlations with four data pairs were considered.

## 3. Results and Discussion

### 3.1. Characterization of the UVC-LED Reactor

The UVC-LED reactor was characterized three times for the maximum driving current of 360 mA using KI/KIO_3_ actinometry: before the first experiment with 1.32 mW∙cm^−2^, after the first three experiments with 1.35 mW∙cm^−2^ and after five runs with 1.12 mW∙cm^−2^ (mean = 1.26 mW∙cm^−2^). With a hydraulic retention time of approximately 1.6 s at a flow of 4.25 L∙h^−1^, this leads to a fluence of 2 mJ∙cm^−2^. Comparing the fluence to the study of Jarvis et al. [112], who reported an inactivation rate of 0.171 cm^2^∙mJ^−1^ for MS2 phages using a 275 nm LED with an irradiance of 4.9 mW∙cm^−2^, this would result in a log removal of 0.35. The applied fluence of 2 mJ∙cm^−2^ is 10 or more times lower compared to the fluences applied for UV disinfection as biofouling control previously reported in the literature [58,59,60,61]. For a setting of 180 mA, no actinometry was performed. However, reducing the current from 360 to 180 mA, according to the datasheet of the LED [67], would result in approximately 42% of the irradiation power (0.85 mJ∙cm^−2^).

### 3.2. Impact of UVC Pretreatment on the Biofilm Formation and Hydraulic Resistance

**Figure 3:**
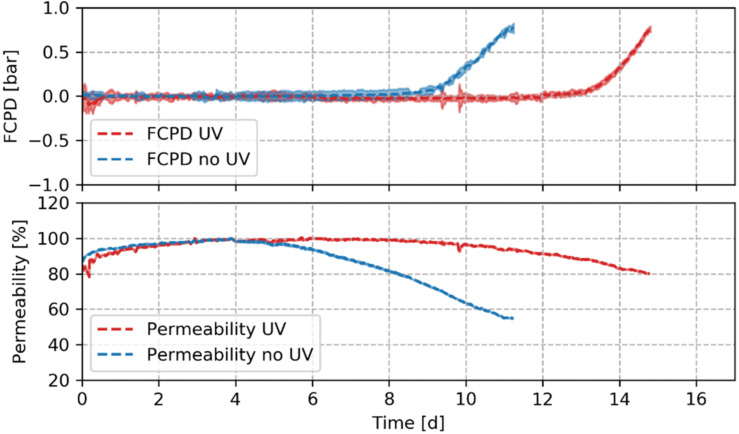
Representative mean feed channel pressure drop (FCPD) increase and mean relative permeability drop over time with and without UV in situ pretreatment for one experiment. Shaded areas represent the 95% confidence intervals. Mean value and confidence intervals are calculated for 1.5 h time period.

Seven successful biofouling experiments were performed—six at a fluence of 2 mJ∙cm^−2^, one at 0.85 mJ∙cm^−2^. A representative time series of the FCPD increase and a drop of the relative membrane permeability after compaction for one of the experiments is depicted in Figure 3. The other biofouling experiments performed showed similar curves (data not shown). The time for the biofilm growth for biofouling experiments is comparable to times reported in the literature [113]. The initial increase of permeability is likely to be caused by the storage in NaHSO_3_ and H_2_O_2_. This increase is similar as reported by Harif et al. [58], who linked it to modified surface properties of the membrane, as they are sensitive to cleaning agents [114]. Nevertheless, no detailed explanation was given. Within the time series of the FCPD increase and permeability decline, a clear distinction between the UV treated and reference line can be seen.

In total, six sets of experiments using the maximum LED driving current are compared for the delay in biofilm formation and the impact of UV irradiation on the hydraulic resistance (Table 3). On average, the membrane filtration experiment with UV pretreatment reached an FCPD of 0.8 bar two days later, which equals 16.5% compared to the non-treated experiment. The drop in hydraulic resistance of the fouling layer, on average, was 3.4 × 10^13^ m^−1^ in the experiments receiving a UV treatment. Thus, on average, the hydraulic resistance of the fouling layer was reduced by 48.8% in a pairwise comparison. Only in one experiment (experiment 5), no reduced hydraulic resistance was observed; on the contrary, the UV-irradiated biofilm exhibited an increase by 18%. The delay in biofilm formation, as well as the change in biofilm resistance, is still significant (*p* < 0.05) for a one tailed non-parametric Wilcoxon signed-rank test. Thus, the UV pretreatment (2 mJ∙cm^−2^) seems to significantly delay the biofilm formation, as well as reducing the hydraulic resistance of the formed fouling layer in a severe biofouling state.

For the eighth experiment, the driving current was reduced to 180 mA (0.85 mJ∙cm^−2^) to investigate if there is a certain threshold of UV fluence for a successful biofouling control. Interestingly, in experiment 8, no delay in biofilm build-up was observed (time difference 5 h), but the resistance of the UV-treated biofilm was only 50% of the control. The fluence of 0.85 mJ∙cm^−2^ does not seem to be enough for delaying the biofilm formation. Hence, it is assumed that the needed fluence for effective biofouling control is up to 2 mJ∙cm^−2^ for the given UVC-LED reactor.

In total three mechanism are supposed to explain the observed delayed biofilm build-up due to the in situ UV pretreatment:

1. Reduction of viable bacteria in the feed: UV disinfection using UVC light is a process known for its capability to inactivate microorganisms [45,46,47,48]. Inactivation was observed in studies investigating the potential of UV pretreatment for biofouling control [58,60,61]. Li et al. [115] reported that during UV disinfection of *Escherichia coli*, irradiation using a 278 nm wavelength LED inhibited photoreactivation and dark repair, probably caused by impairment of protein activities.
2. Changed adhesion properties: RO membranes are commonly negatively charged and rather hydrophobic at pH 7 and becoming more hydrophilic with increasing ion concentrations [116]. During UVA disinfection a depolarization of the cells’ membrane potential was observed [56]. Similar effects are assumed to happen while using 278 nm UV-LEDs, with potential effects on the proteins of the cells [115]. On the one hand, a reduction in cell membrane potential would lead to a lower electric repulsion with the membrane which could lead to better adhesion. On the other hand, according to Otto and Silhavy [117], the outer membrane lipoprotein NlpE is required for activation of the cpxR system and a successful attachment of cells to hydrophobic surfaces. In case the NlpE protein of the cell membrane is damaged due to the UV treatment, it could possibly lead to a reduced adhesion. However, the CpxRA system is complex and can be affected by multiple causes [118,119]. Whereas Kolappan and Satheesh [62] observed a reduced attachment of *Alteromonas* sp. cells to hard surfaces due to UV treatment, Friedman et al. [120] rather linked a reduced viable cell count to a reduced cell abundance on surfaces within their experiments.
3. Cell cycle arrest: Cells experiencing DNA damage might delay or stop the cell cycle to provide more time for DNA repair [57,121]. This delay could further impact the biofilm build-up.

Whereas a change in adhesion properties remains unclear, a reduction of the viable bacteria in the feed and a delay or arrest of the cell cycle seems likely to cause the observed delay in biofouling.

### 3.3. Membrane Autopsy and Biofilm Analysis

**Figure 4:**
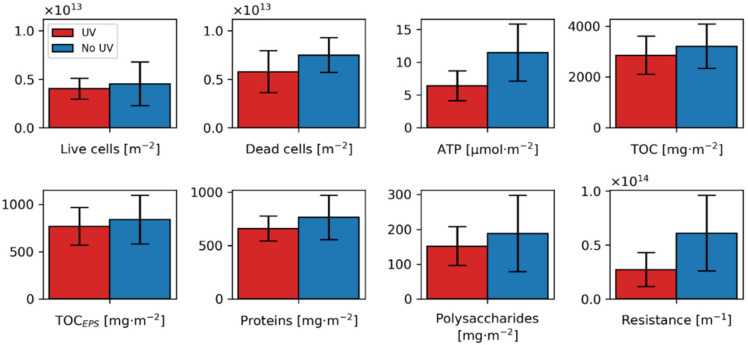
Summary biofilm parameter. Bar plots showing 95% confidence intervals following a t-distribution (*n* = 6). Biofilm resistance is plotted for biofilms with a conducted biofilm analysis (*n* = 5).

Differences in biofilm composition with and without UV treatment were examined by determining the number of live and dead cells as well as ATP and TOC contents in the biofilm at the end of the biofouling experiment (n = 6). The results for the parameters analyzed are summarized in Figure 4. Values for ATP, TDC and TOC are comparable to values reported in the literature for biofouling [15] (pp. 28, 250). The same is true for proteins and polysaccharides [122]. On average, ATP/TDC values were 3.3 and 4.9 × 10^−16^ g per cell for UV treated and untreated biofilms, respectively. Those are comparable to the values found in the literature [123,124]. ATP/TDC_live_ was found to be 8.5 and 15.6 × 10^−16^ g per cell for UV pretreated and untreated biofilms. For the analyzed parameters, a one-tailed Wilcoxon signed-rank test revealed four significant differences. First, the ATP levels seem to be lower in the UV pretreated biofilms (*p* = 0.016, W = 0). Second, the cell count for cells with membrane damage, referred to as dead cells, is higher without pretreatment (*p* = 0.031, W = 1), whereas the cells without cell damage did not seem to differ significantly (*p* = 0.422, W = 9). TDC in general was reduced (*p* = 0.047, W = 2). As a consequence, the ATP/TDC ratio was also reduced by more than 30% through the UV treatment (*p* = 0.031, W = 1). TOC, proteins and polysaccharides were reduced on average, but no statistical significance could be revealed. In summary, all of the measured parameters were reduced by UV treatment at the same degree of biofouling (FCPD = 0.8 bar). The results of the biofilm analyses support the observation of a reduced biofilm resistance.

### 3.4. Fluorescence Spectroscopy and PARAFAC Modeling

**Figure 5:**
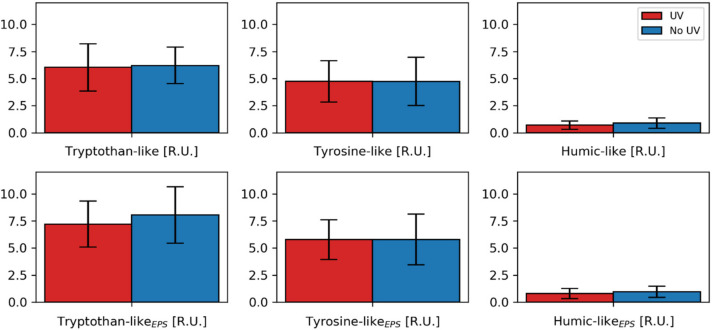
Maximum fluorescence intensity (F_max_) for the modeled components. Bar plots with 95% confidence intervals following a t-distribution (*n* = 6).

A three-component PARAFAC model was built from the fluorescence spectroscopy analysis of the biofilm samples. Details are reported in the Supplementary Materials (Figure S4) [68]. According to its emission and excitation spectrum, Component 1 (C1) could be identified as tryptophan-like (protein) [125,126]. Component 2 (C2) could be characterized as tyrosine-like (protein) [127,128] and component 3 (C3) as humic-like of terrestrial origin [126,129]. For both the filtered and the EPS sample, the maximum fluorescence intensity (F_max_) was very similar (Figure 5). The highest F_max_ values were observed for tryptophan- and tyrosine-like proteins. Nevertheless, it needs to be considered that due to possible differences in the quantum yield of the different components, the calculated F_max_ values cannot be linked directly to a difference in concentration [130]. Still, for none of the modeled components, a significant difference for the UV pretreated biofilm was revealed. This indicates that the composition of the protein- and humic-like constituents is not changed, or at least no change can be monitored with fluorescence spectroscopy. Perhaps an analysis with LC-OCD as performed in the study of Elhadidy et al. [123] could reveal differences in the molecular size distribution of the EPS.

### 3.5. 16S rRNA Amplicon Sequencing

**Figure 6:**
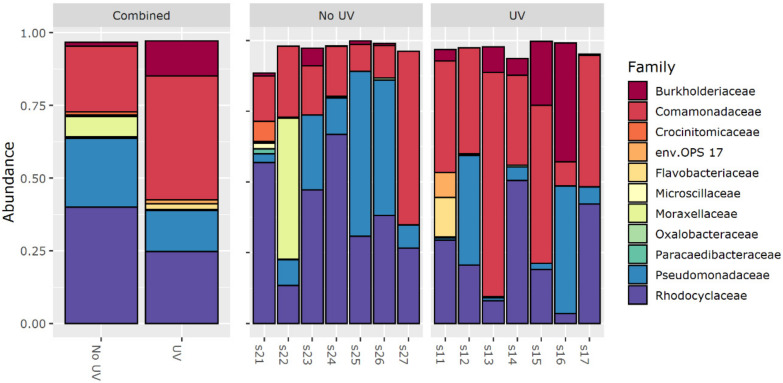
Overview of the relative abundance of the families identified in the treated and non-treated samples. The first number in the sample name represents the treatment condition (2 = untreated, 1 = treated), whereas the second number represents the experimental run. As only the 40 most dominant amplicon sequence variants (ASVs) are plotted, an abundance of 1 is not reached completely.

16S rRNA amplicon sequencing was used to explore the microbial community in the formed biofilms. An overview of the bacteria families present (considering the 40 most dominant amplicon sequence variants (ASVs)) in the treated and non-treated samples is given in Figure 6. Based on these results, the families Rhodocyclaceae, Pseudomonadaceae, Comamonadaceae and Burkholderiaceae dominate the microbial community. Especially the abundance of Burkholderiaceae and Pseudomonadacea has been observed frequently on membrane biofilms [21,28]. For all samples, the dominating class is Gammaproteobacteria, but it shall be noted that according to a recent study of Parks et al. [131], Betaproteobacteria are now reclassified as an order within the class of Gammaproteobacteria (Burkholderiales [132]). A distance-based redundancy analysis differentiating different treatment conditions is presented in the Supplementary Materials (Figure S5) [68]. A distinction between the treated and untreated samples was not significant after applying an ANOVA test (*p* = 0.074, F = 2.122). However, analyzing the variance using PERMANOVA and Bray Curtis distance matrices considering different experimental runs (Adonis ~ treatment + run), a significant difference for the runs (*p* = 0.006, F = 1.918) and treatment (*p* = 0.021, F = 2.154) was detected. Overall, the microbial community of the biofilms seems to differ.

After investigating the differences in the community composition in general, the DESeq2 analysis was used to determine changes at the ASV level. In total, a difference for five ASVs/species was found (*p* adjusted < 0.05). Their base mean, log 2 fold change and adjusted *p*-value are given in Table 4. On the one hand *Novosphingobium* sp., *Acidovorax* sp., *Sphingomonas* sp. and *Ralstonia* sp. show an increased abundance within the UV pretreated biofilms. On the other hand, *Delftia* sp., if present, seems to be reduced by UV treatment. The differences in abundance could be explained as the genera being rather UV resistant or UV sensitive. This could be caused by a different GC content in the genomes [50,51,52] or other biological responses to UV light.

### 3.6. Correlation of Hydraulic Resistance to the Main Biofilm Attributes

**Figure 7:**
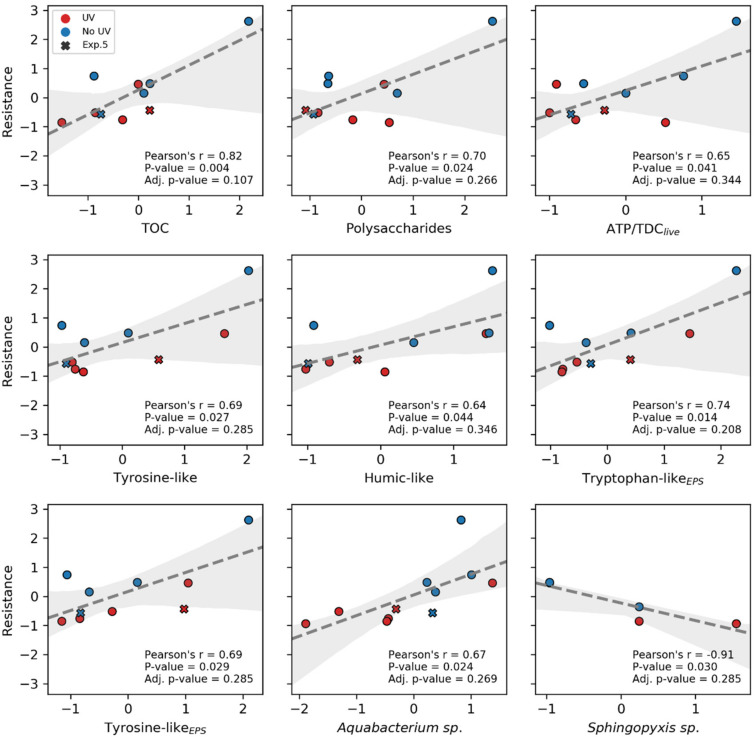
Correlations with biofilm resistance, shaded area representing the 95% confidence intervals. As the values are transformed, no units are depicted on the axis. The x markers in the graphs represent experiment 5, for which no effect of UV treatment for the resistance could be revealed.

To further elucidate the coherences of the hydraulic resistance of the biofilms with the attributes analyzed, a correlation analysis according to Pearson was performed. To illustrate the coherences with the biofilm resistance, correlations with a *p* value of less than 0.05 and a correlation coefficient larger than 0.5 are summarized in Figure 7. A good correlation between TOC, polysaccharides, ATP content normalized to the cells without membrane damage (ATP/TDC_live_) as well as for the humic- and protein-like components was found. Whereas *Sphingopyxis* sp. seem to decrease with hydraulic resistance, the genus of *Aquabacterium* sp. is found in an increased abundance at higher resistances. *Aquabacterium* was found in biofilms of drinking water distribution systems [133] and the study of Gao et al. [134] recognized an increasing presence of *Aquabacterium* at temperatures of 20 and 10 °C. Nonetheless, to the author’s knowledge there is no further information about the EPS production of *Aquabacterium* and its role in increasing hydraulic resistance within biofilms. Nevertheless, parameters showing a correlation with the resistance were not affected significantly by UV treatment. Significant differences were monitored for ATP-levels, TDC and TDC_dead_, as well as for the ATP content normalized to the TDC in total (ATP/TDC). However, ATP/TDC and ATP/TDC_live_ are assumed to be highly correlated (r = 0.797, *p* = 0.002) and as a difference in ATP/TDC was observed for the treated biofilms, it is assumed that ATP/TDC or ATP/TDC_live_ could indicate the mechanisms behind a changed hydraulic resistance of the biofilms. Furthermore, it should be mentioned that when excluding the analysis of experiment 5, a non-parametric one-tailed Mann-Whitney-U-test showed a significant difference for the ATP/TDC_live_ content comparing the treated and the non-treated groups in general (*p* = 0.018, U = 2). Consequently, it seems that the ATP/TDC_live_ ratio is linked to the resistance and is responsible for the difference observed by UV treatment.

Even though differences in the biofilm composition and correlations for the resistance were detected, the mechanisms behind the change in biofilm attributes are complex. Some key ideas that could be helpful for future research are discussed in the following:

1. Different quantity in EPS production: As reported in the literature [40,122], polysaccharides and proteins are linked to hydraulic resistance. Even though no significant change for EPS content of treated and untreated biofilms could be revealed, a consistent trend for a reduced average in EPS and TOC quantities for the UV pretreated biofilm could be seen (11, 8, 14 and 19% for TOC, TOC_EPS_, Proteins and Polysaccharides, respectively). A significant difference in EPS quantity could probably not be detected due to the low number of replicates (n = 6) or because there exists an interplay among those parameters, making a simple comparison difficult.
2. Changed EPS composition/quality and biofilm morphology due to changed biodiversity: UV pretreatment seems to alter the microbial community present in biofilms. Interestingly, the genera that showed a significantly differential abundance did not correlate with the biofilm resistance. Furthermore, within the PARAFAC analysis, at least no differences for expression of tyrosine- or tryptophan-like proteins was found. Still, a change in the microbial community could lead to different EPS quality [20]. A difference in EPS composition could also be polysaccharide-related or not be detected with fluorescence measurements.
3. Reduced ATP levels impact quorum sensing or vice versa: The ATP/TDC_live_ ratio correlates with the hydraulic resistance and seems to be changed by the UV treatment. Still the cause for a reduced ATP level and the effects for the biofilm resistance are not clear. Yang et al. [135] observed an ATP reduction over time for UV-irradiated bacteria, especially for medium pressure UV lamps. Thus, cells irradiated and subsequently adhered to the biofilm could exhibit lower ATP levels. Furthermore, different kinds of microorganisms could show different levels of ATP [136]. Jiang and Liu [137] recognized that if ATP production is limited in aerobic granules, the amount of AHL messenger molecules and EPS is reduced. Vice versa, Zhang et al. [138] showed in their study that with increasing concentration of quorum sensing molecules (AHLs), ATP levels rose. The reduced ATP levels in the UV treated biofilms could, therefore, indicate less quorum sensing. Additionally, Zhang et al. [138] observed that ATP needs to be present in a sufficient amount for EPS production. A reduction of quorum sensing could also be caused by an altered microbial community [39,138,139]. Not only microorganisms showing increased AHL production could show a changed abundance, but also those quenching AHL signals. According to the review of Uroz et al. [39], *Delftia acidovorans* [140], *Ralstonia sp.XJ12B* [141] and *Sphingomonas* [142] could degrade or modify AHL signal molecules. Whereas a *Delftia* strain was found with reduced abundance in the UV treated biofilms, the *Ralstonia* and *Sphingomonas* strains were increased. Unfortunately, in this study, it was beyond the scope to directly elucidate how the AHL concentration affects the ATP levels. Nevertheless, AHL expression might not only impact the quantity of EPS but also correlate with the production of tightly and loosely bound EPS [139]. This could possibly affect the resistance of the formed biofilm.
4. Introduction of prophage: Stress conditions, such as DNA damage, are known to lead to prophage induction to biofilms, which can further lead to biofilm dispersal or disruption [118,143]. Biofilm dispersal could not only lead to a delayed build-up but also create a more open structure, leading to lower resistance.

In summary, especially the change of microbial community and ATP/TDC_live_ levels (for the same FCPD) are likely to impact the biofilm structure, but could also add to the delay of biofilm formation. The reasons for reduced ATP/TDC_live_ might be linked to a modified diversity or the effects of the UV treatment itself. Nevertheless, ATP is seen as an indicator for active biomass in general [78] [15] (p. 26).

## 4. Conclusions

Findings of this study reveal that UV pretreatment using UVC-LEDs in the immediate vicinity of RO membrane surfaces is capable of delaying biofouling already at low fluences. Furthermore, at the same degree of biofouling (same FCPD) the hydraulic resistance of the biofilms is reduced. Inactivation of the cells in the feed stream, a change in the adhesion properties and an induced cell cycle arrest are suspected to cause the delay of biofilm formation. Evidence was found that the hydraulic resistance might be affected by a modified microbial diversity, EPS composition, ATP levels per cells and possibly also quorum sensing. Nevertheless, the mechanisms behind the delayed and changed biofilm formation could not be elucidated with certainty. Further research is needed, especially regarding quorum sensing with respect to ATP and microbial community, to provide more insights into the underlying mechanisms. Analysis of the biofilm structure using confocal laser scanning microscopy or optical coherence tomography would be very helpful to further evaluate the observed effects or stated hypotheses. Measurements of hydrophilicity could contribute to a deeper understanding of the changed biofilm resistance or adhesion properties. In addition, it should be investigated if the observed effects translate to full-scale application over multiple cleaning cycles and several membrane modules in series. In conclusion, the results of the study suggest that in situ pretreatment of the feed stream, using UVC-LEDs in a flexible reactor design (possibly integrating the LEDs in situ in the pressure vessels) could be a promising environmentally friendly technology for biofouling control in RO and other systems.

## Figures and Tables

**Table 1:** Analyzed biofilm parameters representing different biofilm attributes.

Parameter	Biofilm Property
TOC/TOCEPS	Total organic mass/Total EPS
ATP	Active biomass [78] [15] (p. 26)
Total direct cell counts (TDC)	Dead and living cells
Proteins and polysaccharides	EPS composition
Excitation emission matrix (EEM) using fluorescence spectroscopy	Differentiation tyrosine- and tryptophan-like proteins
16S rRNA amplicon sequencing	Microbial community composition

**Table 2:** Summary of experimental settings.

Experiment No.	Line with UV Treatment	UV Dummy ^1^	LED Current [mA]	Membrane Performance Data	Biofilm Analysis
1	1	Yes	360	Yes	16S
2	1	Yes	360	Yes	All
3	2	Yes	360	No	All
4	1	No	360	Yes	All
5	2	No	360	Yes	All
6	2	No	360	Yes	All
7	1	No	360	Yes, intermittent permeate production	All
8	1	No	180	Yes	No

^1^ Silica glass pipe to ensure same hydraulic retention times in the reference line.

**Table 3:** Summary of membrane performance for six experiments, applying a fluence of 2 mJ∙cm^−2^.

	FCPD Delay	FCPD Delay	Difference Hydraulic Resistance	Difference Hydraulic Resistance
Mean	2.0 d	16.5%	3.4 10^13^ m^−1^	48.8%
95% confidence-t interval	1.0 d	10.0%	2.7 10^13^ ^m-1^	34.9%
p-value one sided Wilcoxon signed-rank test	0.016 *^1^	0.016 *^1^	0.031 *^2^	0.031 *^2^
Wilcoxon test statistic W	21 *^1^	21 *^1^	1 *^2^	1 *^2^

*^1^ Testing for increase compared to UV; *^2^ Testing for reduction compared to UV.

**Table 4:** Summary of DESeq2 results (the arrows in the last column are indicating an increase or reduced abundance due to the UV irradiation).

ASV	Family	Genus	Base Mean	Log 2 Fold Change	p Adjusted	Abundance
45	Sphingomonadaceae	*Novosphingobium*	139	25.8	8 × 10^−17^	↑
46	Comamonadaceae	*Delftia*	101	−24.8	7 × 10^−16^	↓
2	Comamonadaceae	Acidovorax *	21,435	4.0	0.01	↑
60	Sphingomonadaceae	*Sphingomonas*	80	4.5	0.03	↑
4	Burkholderiaceae	*Ralstonia*	5957	3.5	0.03	↑

* identified by BLAST.

## Supplementary Materials

All Supplementary Materials can be found here: https://data.mendeley.com/ doi: 10.17632/rcpkdzzx7s.1 [68].

## References

[1] Mekonnen M.M., Hoekstra A.Y. (2016). Four billion people facing severe water scarcity. Sci. Adv..

[2] Unesco (2017). Wastewater. The Untapped Resource.

[3] Drewes J.E., Khan S., Edzwald J.K. (2011). Water Reuse for Drinking Water Augmentation. Water Quality & Treatment: A Handbook on Drinking Water.

[4] Drewes J.E., Horstmeyer N., Michel P., Khan S., Lema J.M., Suarez Martinez S. (2017). Producing high-quality recycled water. Innovative Wastewater Treatment & Resource Recovery Technologies: Impacts on Energy, Economy and Environment.

[5] Côté P., Siverns S., Monti S. (2005). Comparison of Membrane-based Solutions for Water Reclamation and Desalination. Desalination.

[6] Dolnicar S., Schäfer A.I. (2009). Desalinated versus recycled water: Public perceptions and profiles of the accepters. J. Environ. Manage..

[7] Tomaszewska M. (2007). Industrial wastewater treatment by means of membrane techniques. Polish J. Chem. Technol..

[8] Howe K.J., Hand D.W., Crittenden J.C., Trussell R.R., Tchobanoglous G. (2012). Principles of Water Treatment.

[9] Guo W., Ngo H.-H., Li J. (2012). A mini-review on membrane fouling. Bioresour. Technol..

[10] Koros W.J., Ma Y.H., Shimidzu T. (1996). Terminology for membranes and membrane processes (IUPAC Recommendations 1996). Pure Appl. Chem..

[11] Baker J.S., Dudley L.Y. (1998). Biofouling in membrane systems—A review. Desalination.

[12] Flemming H.-C., Schaule G., Griebe T., Schmitt J., Tamachkiarowa A. (1997). Biofouling—The Achilles heel of membrane processes. Desalination.

[13] Flemming H.-C. (2020). Biofouling and me: My Stockholm syndrome with biofilms. Water Res..

[14] Flemming H.-C., Flemming H.-C., Wingender J., Szewzyk U. (2011). Microbial Biofouling: Unsolved Problems, Insufficient Approaches, and Possible Solutions. Biofilm Highlights.

[15] Vrouwenvelder J.S., Kruithof J., van Loosdrecht M. (2009). Biofouling of Spiral Wound Membrane Systems.

[16] Characklis W.G., Marshall K.C. (1990). Biofilms.

[17] Flemming H.-C., Wingender J., Szewzyk U. (2011). Biofilm Highlights.

[18] Nguyen T., Roddick F.A., Fan L. (2012). Biofouling of water treatment membranes: A review of the underlying causes, monitoring techniques and control measures. Membranes.

[19] Wilbert M.C. (1997). Enhancement of Membrane Fouling Resistance through Surface Modification. A Study Using the Principle of Membrane Fouling and Cleaning To Develop Ways to Enhance Membrane Fouling Resistance.

[20] Flemming H.-C., Wingender J. (2010). The biofilm matrix. Nat. Rev. Microbiol..

[21] Sánchez O. (2018). Microbial diversity in biofilms from reverse osmosis membranes: A short review. J. Membr. Sci..

[22] Khan M.T.O., de Manes C.-L., Aubry C., Gutierrez L., Croue J.P. (2013). Kinetic study of seawater reverse osmosis membrane fouling. Environ. Sci. Technol..

[23] Bereschenko L.A., Heilig G.H.J., Nederlof M.M., van Loosdrecht M.C.M., Stams A.J.M., Euverink G.J.W. (2008). Molecular characterization of the bacterial communities in the different compartments of a full-scale reverse-osmosis water purification plant. Appl. Environ. Microbiol..

[24] Bereschenko L.A., Stams A.J.M., Euverink G.J.W., van Loosdrecht M.C.M. (2010). Biofilm formation on reverse osmosis membranes is initiated and dominated by *Sphingomonas* spp.. Appl. Environ. Microbiol..

[25] Gutman J., Herzberg M., Walker S.L. (2014). Biofouling of reverse osmosis membranes: Positively contributing factors of *Sphingomonas*. Environ. Sci. Technol..

[26] Huang L.-N., de Wever H., Diels L. (2008). Diverse and distinct bacterial communities induced biofilm fouling in membrane bioreactors operated under different conditions. Environ. Sci. Technol..

[27] Pang C.M., Liu W.-T. (2007). Community structure analysis of reverse osmosis membrane biofilms and the significance of Rhizobiales bacteria in biofouling. Environ. Sci. Technol..

[28] De Vries H.J., Stams A.J.M., Plugge C.M. (2020). Biodiversity and ecology of microorganisms in high pressure membrane filtration systems. Water Res..

[29] Herzberg M., Elimelech M. (2008). Physiology and genetic traits of reverse osmosis membrane biofilms: A case study with *Pseudomonas aeruginosa*. ISME J..

[30] Ridgway H.F., Kelly A., Justice C., Olson B.H. (1983). Microbial fouling of reverse-osmosis membranes used in advanced wastewater treatment technology: Chemical, bacteriological, and ultrastructural analyses. Appl. Environ. Microbiol..

[31] Bereschenko L.A., Prummel H., Euverink G.J.W., Stams A.J.M., van Loosdrecht M.C.M. (2011). Effect of conventional chemical treatment on the microbial population in a biofouling layer of reverse osmosis systems. Water Res..

[32] Ayache C., Manes C., Pidou M., Croué J.P., Gernjak W. (2013). Microbial community analysis of fouled reverse osmosis membranes used in water recycling. Water Res..

[33] Kim I.S., Lee J., Kima S.-J., Yu H.-W., Jang A. (2014). Comparative pyrosequencing analysis of bacterial community change in biofilm formed on seawater reverse osmosis membrane. Environ. Technol..

[34] Zodrow K.R., Bar-Zeev E., Giannetto M.J., Elimelech M. (2014). Biofouling and microbial communities in membrane distillation and reverse osmosis. Environ. Sci. Technol..

[35] Ferrera I., Mas J., Taberna E., Sanz J., Sánchez O. (2015). Biological support media influence the bacterial biofouling community in reverse osmosis water reclamation demonstration plants. Biofouling.

[36] Khan M.T., Hong P.-Y., Nada N., Croue J.P. (2015). Does chlorination of seawater reverse osmosis membranes control biofouling?. Water Res..

[37] Tan Y.-J., Sun L.-J., Li B.-T., Zhao X.-H., Yu T., Ikuno N., Ishii K., Hu H.-Y. (2017). Fouling characteristics and fouling control of reverse osmosis membranes for desalination of dyeing wastewater with high chemical oxygen demand. Desalination.

[38] Wingender J., Neu T.R., Flemming H.-C. (1999). Microbial Extracellular Polymeric Substances.

[39] Uroz S., Dessaux Y., Oger P. (2009). Quorum sensing and quorum quenching: The yin and yang of bacterial communication. ChemBioChem.

[40] Dreszer C., Vrouwenvelder J.S., Paulitsch-Fuchs A.H., Zwijnenburg A., Kruithof J.C., Flemming H.-C. (2013). Hydraulic resistance of biofilms. J. Membr. Sci..

[41] Herzberg M., Elimelech M. (2007). Biofouling of reverse osmosis membranes: Role of biofilm-enhanced osmotic pressure. J. Membr. Sci..

[42] Ridgway H.F., Safarik J., Flemming H.-C., Geesey G.G. (1991). Biofouling of reverse osmosis membranes. Biofouling and Biocorrosion in Industrial Water Systems.

[43] Vrouwenvelder J.S., Kruithof J.C., van Loosdrecht M.C.M. (2010). Integrated approach for biofouling control. Water Sci. Technol..

[44] Saad M.A. (1992). Biofouling prevention in RO polymeric membrane systems. Desalination.

[45] Hijnen W.A.M., Beerendonk E.F., Medema G.J. (2006). Inactivation credit of UV radiation for viruses, bacteria and protozoan (oo)cysts in water: A review. Water Res..

[46] Vilhunen S., Särkkä H., Sillanpää M. (2009). Ultraviolet light-emitting diodes in water disinfection. Environ. Sci. Pollut. Res. Int..

[47] Harm W. (1980). Biological Effects of Ultraviolet Radiation, 1. publ.

[48] Soloshenko I.A., Bazhenov V.Y., Khomich V.A., Tsiolko V.V., Potapchenko N.G. (2006). Comparative Research of Efficiency of Water Decontamination by UV Radiation of Cold Hollow Cathode Discharge Plasma Versus That of Low- and Medium-Pressure Mercury Lamps. IEEE Trans. Plasma Sci..

[49] Reed R.H. (2004). The Inactivation of Microbes by Sunlight: Solar Disinfection as a Water Treatment Process. Adv. Appl. Microbiol..

[50] Pullerits K., Ahlinder J., Holmer L., Salomonsson E., Öhrman C., Jacobsson K., Dryselius R., Forsman M., Paul C.J., Rådström P. (2020). Impact of UV irradiation at full scale on bacterial communities in drinking water. NPJ Clean Water.

[51] Reichenberger E.R., Rosen G., Hershberg U., Hershberg R. (2015). Prokaryotic nucleotide composition is shaped by both phylogeny and the environment. Genome Biol. Evol..

[52] Warnecke F., Sommaruga R., Sekar R., Hofer J.S., Pernthaler J. (2005). Abundances, identity, and growth state of actinobacteria in mountain lakes of different UV transparency. Appl. Environ. Microbiol..

[53] McKinney C.W., Pruden A. (2012). Ultraviolet disinfection of antibiotic resistant bacteria and their antibiotic resistance genes in water and wastewater. Environ. Sci. Technol..

[54] Riesenman P.J., Nicholson W.L. (2000). Role of the spore coat layers in Bacillus subtilis spore resistance to hydrogen peroxide, artificial UV-C, UV-B, and solar UV radiation. Appl. Environ. Microbiol..

[55] Mason J.M., Setlow P. (1986). Essential role of small, acid-soluble spore proteins in resistance of Bacillus subtilis spores to UV light. J. Bacteriol..

[56] Berney M., Weilenmann H.-U., Egli T. (2006). Flow-cytometric study of vital cellular functions in *Escherichia coli* during solar disinfection (SODIS). Microbiology.

[57] Matallana-Surget S., Wattiez R. (2013). Impact of Solar Radiation on Gene Expression in Bacteria. Proteomes.

[58] Harif T., Elifantz H., Margalit E., Herzberg M., Lichi T., Minz D. (2011). The effect of UV pre-treatment on biofouling of BWRO membranes: A field study. Desalin. Water Treat..

[59] Martino D., Ahmed H., Veronique H., Cyril M., Ning R.Y. (2011). Assessment of UV Pre-Treatment to Reduce Fouling of NF Membranes. Expanding Issues in Desalination.

[60] Marconnet C., Houari A., Seyer D., Djafer M., Coriton G., Heim V., Di Martino P. (2011). Membrane biofouling control by UV irradiation. Desalination.

[61] Otaki M., Takizawa S., Ohgaki S. (1998). Control and modeling of membrane fouling due to microorganism growth by UV pretreatment. Water Sci. Technol..

[62] Kolappan A., Satheesh S. (2011). Efficacy of UV Treatment in the Management of Bacterial Adhesion on Hard Surfaces. Pol. J. Microbiol..

[63] Lakretz A., Mamane H., Asa E., Harif T., Herzberg M. (2018). Biofouling control by UV/H_2_O_2_ pretreatment for brackish water reverse osmosis process. Environ. Sci. Water Res. Technol..

[64] Matin A., Khan Z., Zaidi S.M.J., Boyce M.C. (2011). Biofouling in reverse osmosis membranes for seawater desalination: Phenomena and prevention. Desalination.

[65] Song K., Mohseni M., Taghipour F. (2016). Application of ultraviolet light-emitting diodes (UV-LEDs) for water disinfection: A review. Water Res..

[66] Würtele M.A., Kolbe T., Lipsz M., Külberg A., Weyers M., Kneissl M., Jekel M. (2011). Application of GaN-based ultraviolet-C light emitting diodes--UV LEDs--for water disinfection. Water Res..

[67] (2018). Laser Components GmbH. 278 nm 100 mW 6060 1in1 Flat LED PKG. https://www.google.com/url?sa=t&rct=j&q=&esrc=s&source=web&cd=1&ved=2ahUKEwj7toXy_tPoAhXusaQKHbluDOsQFjAAegQIBhAB&url=https%3A%2F%2Fwww.lasercomponents.com%2Ffileadmin%2Fuser_upload%2Fhome%2FDatasheets%2Flg%2Fleuva66h70hf00_278nm_high_power.pdf&usg=AOvVaw0lro6w8EigE-S1HTUS-UCV.

[68] Sperle P., Wurzbacher C., Drewes J.E., Skibinski B. (2020). Supplementary Materials: Reducing the Impacts of Biofouling in RO Membrane Systems through in-situ Low Fluence Irradiation Employing UVC-LEDs [Dataset], Mendeley Data, V1. https://data.mendeley.

[69] Vrouwenvelder J.S., von der Graf Schulenburg D.A., Kruithof J.C., Johns M.L., van Loosdrecht M.C.M. (2009). Biofouling of spiral-wound nanofiltration and reverse osmosis membranes: A feed spacer problem. Water Res..

[70] Radu A.I., Vrouwenvelder J.S., van Loosdrecht M.C.M., Picioreanu C. (2010). Modeling the effect of biofilm formation on reverse osmosis performance: Flux, feed channel pressure drop and solute passage. J. Membr. Sci..

[71] DuPont (2020). FilmTec™ Reverse Osmosis Membranes. TechnicalManual. https://www.dupont.com/content/dam/dupont/amer/us/en/water-solutions/public/documents/en/45-D01504-en.pdf.

[72] Li K., Li S., Huang T., Dong C., Li J., Zhao B., Zhang S. (2019). Chemical Cleaning of Ultrafiltration Membrane Fouled by Humic Substances: Comparison between Hydrogen Peroxide and Sodium Hypochlorite. Int. J. Environ. Res. Public Health.

[73] Ling R., Yu L., Pham T.P.T., Shao J., Chen J.P., Reinhard M. (2017). The tolerance of a thin-film composite polyamide reverse osmosis membrane to hydrogen peroxide exposure. J. Membr. Sci..

[74] Kucera J. (2019). Biofouling of Polyamide Membranes: Fouling Mechanisms, Current Mitigation and Cleaning Strategies, and Future Prospects. Membranes.

[75] Dow (2020). FILMTEC™ Reverse Osmosis Membranes. Technical Manual Form. https://www.rainmandesal.com/wp-content/uploads/2018/09/dow-filmtec-sw30-manual.pdf.

[76] Matar G., Gonzalez-Gil G., Maab H., Nunes S., Le-Clech P., Vrouwenvelder J., Saikaly P.E. (2016). Temporal changes in extracellular polymeric substances on hydrophobic and hydrophilic membrane surfaces in a submerged membrane bioreactor. Water Res..

[77] Han X., Wang Z., Zhu C., Wu Z. (2013). Effect of ultrasonic power density on extracting loosely bound and tightly bound extracellular polymeric substances. Desalination.

[78] Holm-Hansen O., Booth C.R. (1966). The measurement of adenosine triphosphate in the ocean and its ecological significance1. Limnol. Oceanogr..

[79] (2019). DIN EN 1484:2019-04, Wasseranalytik_- Anleitungen zur Bestimmung des Gesamten Organischen Kohlenstoffs_(TOC) und des Gelösten Organischen Kohlenstoffs_(DOC).

[80] Promega Corporation (2016). BacTiter-Glo™ Microbial Cell Viability Assay.

[81] Boulos L., Prévost M., Barbeau B., Coallier J., Desjardins R. (1999). LIVE/DEAD^®^ BacLight™: Application of a new rapid staining method for direct enumeration of viable and total bacteria in drinking water. J. Microbiol. Methods.

[82] Masuko T., Minami A., Iwasaki N., Majima T., Nishimura S.-I., Lee Y.C. (2005). Carbohydrate analysis by a phenol-sulfuric acid method in microplate format. Anal. Biochem..

[83] (2009). DIN EN ISO 10304-1:2009-07, Wasserbeschaffenheit_- Bestimmung von Gelösten Anionen Mittels Flüssigkeits-Ionenchromatographie_- Teil_1: Bestimmung von Bromid, Chlorid, Fluorid, Nitrat, Nitrit, Phosphat und Sulfat (ISO_10304-1:2007); Deutsche Fassung EN_ISO_10304-1:2009.

[84] (2000). DIN EN ISO 7980:2000-07, Wasserbeschaffenheit_- Bestimmung von Calcium und Magnesium_- Verfahren mittels Atomabsorptionsspektrometrie (ISO_7980:1986); Deutsche Fassung EN_ISO_7980:2000.

[85] (1998). DIN 38406-6:1998-07, Deutsche Einheitsverfahren zur Wasser-, Abwasser- und Schlammuntersuchung_- Kationen (Gruppe_E)_- Teil_6: Bestimmung von Blei mittels Atomabsorptionsspektrometrie (AAS) (E_6).

[86] (2011). DIN 38405-9:2011-09, Deutsche Einheitsverfahren zur Wasser-, Abwasser- und Schlammuntersuchung_- Anionen (Gruppe_D)_- Teil_9: Photometrische Bestimmung von Nitrat_(D_9).

[87] (2004). DIN EN ISO 6878:2004-09, Wasserbeschaffenheit_- Bestimmung von Phosphor_- Photometrisches Verfahren Mittels Ammoniummolybdat (ISO_6878:2004); Deutsche Fassung EN_ISO_6878:2004.

[88] Rahn R.O. (1997). Potassium Iodide as a Chemical Actinometer for 254 nm Radiation: Use of lodate as an Electron Scavenger. Photochem. Photobiol..

[89] Rahn R.O., Stefan M.I., Bolton J.R., Goren E., Shaw P.-S., Lykke K.R. (2003). Quantum Yield of the Iodide–Iodate Chemical Actinometer: Dependence on Wavelength and Concentration. Pure Appl. Chem..

[90] Zou X.-Y., Lin Y.-L., Xu B., Cao T.-C., Tang Y.-L., Pan Y., Gao Z.-C., Gao N.-Y. (2019). Enhanced inactivation of *E. coli* by pulsed UV-LED irradiation during water disinfection. Sci. Total Environ..

[91] Wang W.-L., Wu Q.-Y., Li Z.-M., Lu Y., Du Y., Wang T., Huang N., Hu H.-Y. (2017). Light-emitting diodes as an emerging UV source for UV/chlorine oxidation: Carbamazepine degradation and toxicity changes. Chem. Eng. J..

[92] Ranga Suri N.N.R., Murty M.N., Athithan G., Ranga Suri N.N.R., Murty M.N., Athithan G. (2019). Outlier Detection. Outlier Detection: Techniques and Applications.

[93] Murphy K.R., Stedmon C.A., Graeber D., Bro R. (2013). Fluorescence spectroscopy and multi-way techniques. PARAFAC. Anal. Methods.

[94] Murphy K.R., Stedmon C.A., Wenig P., Bro R. (2014). OpenFluor—An online spectral library of auto-fluorescence by organic compounds in the environment. Anal. Methods.

[95] Bro R., Kiers H.A.L. (2003). A new efficient method for determining the number of components in PARAFAC models. J. Chemom..

[96] Stedmon C.A., Bro R. (2008). Characterizing dissolved organic matter fluorescence with parallel factor analysis: A tutorial. Limnol. Oceanogr. Methods.

[97] Callahan B.J., McMurdie P.J., Rosen M.J., Han A.W., Johnson A.J.A., Holmes S.P. (2016). DADA2: High-resolution sample inference from *Illumina amplicon* data. Nat. Methods.

[98] Wright E.S. (2016). Using DECIPHER v2.0 to Analyze Big Biological Sequence Data in R. R J..

[99] Zhang Z., Schwartz S., Wagner L., Miller W. (2000). A greedy algorithm for aligning DNA sequences. J. Comput. Biol..

[100] Altschul S.F., Gish W., Miller W., Myers E.W., Lipman D.J. (1990). Basic local alignment search tool. J. Mol. Biol..

[101] Karsch-Mizrachi I., Takagi T., Cochrane G. (2018). The international nucleotide sequence database collaboration. Nucleic Acids Res..

[102] Heck K.L., van Belle G., Simberloff D. (1975). Explicit Calculation of the Rarefaction Diversity Measurement and the Determination of Sufficient Sample Size. Ecology.

[103] Hurlbert S.H. (1971). The Nonconcept of Species Diversity: A Critique and Alternative Parameters. Ecology.

[104] Legendre P., Anderson M.J. (1999). Distance-Based Redundancy Analysis: Testing Multispecies Responses in Multifactorial Ecological Experiments. Ecol. Monogr..

[105] McArdle B.H., Anderson M.J. (2001). Fitting Multivariate Models to Community Data: A Comment on Distance-Based Redundancy Analysis. Ecology.

[106] Oksanen J., Blanchet F.G., Friendly M., Kindt R., Legendre P., McGlinn D., Minchin P.R., O’Hara R.B., Simpson G.L., Solymos P. (2019). Vegan: Community Ecology Package. https://CRAN.R-project.org/package=vegan.

[107] Anderson M.J. (2001). A new method for non-parametric multivariate analysis of variance. Austral. Ecol..

[108] Love M.I., Huber W., Anders S. (2014). Moderated estimation of fold change and dispersion for RNA-seq data with DESeq2. Genome Biol..

[109] Stephens M. (2017). False discovery rates: A new deal. Biostatistics.

[110] Benjamini Y., Hochberg Y. (1995). Controlling the False Discovery Rate: A Practical and Powerful Approach to Multiple Testing. J. R. Stat. Soc. Series B.

[111] Lagkouvardos I., Fischer S., Kumar N., Clavel T. (2017). Rhea: A transparent and modular R pipeline for microbial profiling based on 16S rRNA gene amplicons. PeerJ.

[112] Jarvis A., Goslan H. (2019). Application of Ultraviolet Light-Emitting Diodes (UV-LED) to Full-Scale Drinking-Water Disinfection. Water.

[113] Farhat N.M., Vrouwenvelder J.S., van Loosdrecht M.C.M., Bucs S.S., Staal M. (2016). Effect of water temperature on biofouling development in reverse osmosis membrane systems. Water Res..

[114] Subramani A., Hoek E.M.V. (2010). Biofilm formation, cleaning, re-formation on polyamide composite membranes. Desalination.

[115] Li G.-Q., Wang W.-L., Huo Z.-Y., Lu Y., Hu H.-Y. (2017). Comparison of UV-LED and low pressure UV for water disinfection: Photoreactivation and dark repair of *Escherichia coli*. Water Res..

[116] Hurwitz G., Guillen G.R., Hoek E.M.V. (2010). Probing polyamide membrane surface charge, zeta potential, wettability, and hydrophilicity with contact angle measurements. J. Membr. Sci..

[117] Otto K., Silhavy T.J. (2002). Surface sensing and adhesion of *Escherichia coli* controlled by the Cpx-signaling pathway. Proc. Natl. Acad. Sci. USA..

[118] Landini P. (2009). Cross-talk mechanisms in biofilm formation and responses to environmental and physiological stress in *Escherichia coli*. Res. Microbiol..

[119] Dorel C., Lejeune P., Rodrigue A. (2006). The Cpx system of *Escherichia coli*, a strategic signaling pathway for confronting adverse conditions and for settling biofilm communities?. Res. Microbiol..

[120] Friedman L., Harif T., Herzberg M., Mamane H. (2016). Mitigation of Biofilm Colonization on Various Surfaces in a Model Water Flow System by Use of UV Treatment. Water Air Soil Pollut..

[121] Friedberg E.C. (2003). DNA damage and repair. Nature.

[122] Desmond P., Best J.P., Morgenroth E., Derlon N. (2018). Linking composition of extracellular polymeric substances (EPS) to the physical structure and hydraulic resistance of membrane biofilms. Water Res..

[123] Elhadidy A.M., van Dyke M.I., Chen F., Peldszus S., Huck P.M. (2017). Development and application of an improved protocol to characterize biofilms in biologically active drinking water filters. Environ. Sci. Water Res. Technol..

[124] Velten S., Hammes F., Boller M., Egli T. (2007). Rapid and direct estimation of active biomass on granular activated carbon through adenosine tri-phosphate (ATP) determination. Water Res..

[125] Yamashita Y., Panton A., Mahaffey C., Jaffé R. (2011). Assessing the spatial and temporal variability of dissolved organic matter in Liverpool Bay using excitation–emission matrix fluorescence and parallel factor analysis. Ocean Dyn..

[126] Coble P.G. (1996). Characterization of marine and terrestrial DOM in seawater using excitation-emission matrix spectroscopy. Mar. Chem..

[127] Yamashita Y., Boyer J.N., Jaffé R. (2013). Evaluating the distribution of terrestrial dissolved organic matter in a complex coastal ecosystem using fluorescence spectroscopy. Cont. Shelf Res..

[128] Yamashita Y., Tanoue E. (2004). Chemical characteristics of amino acid-containing dissolved organic matter in seawater. Org. Geochem..

[129] Painter S.C., Lapworth D.J., Woodward E.M.S., Kroeger S., Evans C.D., Mayor D.J., Sanders R.J. (2018). Terrestrial dissolved organic matter distribution in the North Sea. Sci. Total Environ..

[130] Baghoth S.A., Sharma S.K., Amy G.L. (2011). Tracking natural organic matter (NOM) in a drinking water treatment plant using fluorescence excitation-emission matrices and PARAFAC. Water Res..

[131] Parks D.H., Chuvochina M., Waite D.W., Rinke C., Skarshewski A., Chaumeil P.-A., Hugenholtz P. (2018). A standardized bacterial taxonomy based on genome phylogeny substantially revises the tree of life. Nat. Biotechnol..

[132] The SILVA Ribosomal RNA Database Project The SILVA Taxonomy. https://www.arb-silva.de/documentation/silva-taxonomy/.

[133] Kalmbach S. (2000). In situ probing reveals Aquabacterium commune as a widespread and highly abundant bacterial species in drinking water biofilms. Water Res..

[134] Gao D.-W., Wen Z.-D., Li B., Liang H. (2013). Membrane fouling related to microbial community and extracellular polymeric substances at different temperatures. Bioresour. Technol..

[135] Yang C., Sun W., Ao X. (2020). Bacterial inactivation, DNA damage, and faster ATP degradation induced by ultraviolet disinfection. Front. Environ. Sci. Eng..

[136] Ivanova E.P., Alexeeva Y.V., Pham D.K., Wright J.P., Nicolau D.V. (2006). ATP level variations in heterotrophic bacteria during attachment on hydrophilic and hydrophobic surfaces. Int. Microbiol. Off. J. Span. Soc. Microbiol..

[137] Jiang B., Liu Y. (2013). Dependence of structure stability and integrity of aerobic granules on ATP and cell communication. Appl. Microbiol. Biotechnol..

[138] Zhang Z., Cao R., Jin L., Zhu W., Ji Y., Xu X., Zhu L. (2019). The regulation of N-acyl-homoserine lactones (AHLs)-based quorum sensing on EPS secretion via ATP synthetic for the stability of aerobic granular sludge. Sci. Total Environ..

[139] Ma H., Wang X., Zhang Y., Hu H., Ren H., Geng J., Ding L. (2018). The diversity, distribution and function of N-acyl-homoserine lactone (AHL) in industrial anaerobic granular sludge. Bioresour. Technol..

[140] Jafra S., Przysowa J., Czajkowski R., Michta A., Garbeva P., van der Wolf J.M. (2006). Detection and characterization of bacteria from the potato rhizosphere degrading N-acyl-homoserine lactone. Can. J. Microbiol..

[141] Lin Y.-H., Xu J.-L., Hu J., Wang L.-H., Ong S.L., Leadbetter J.R., Zhang L.-H. (2003). Acyl-homoserine lactone acylase from Ralstonia strain XJ12B represents a novel and potent class of quorum-quenching enzymes. Mol. Microbiol..

[142] D’Angelo-Picard C., Faure D., Penot I., Dessaux Y. (2005). Diversity of N-acyl homoserine lactone-producing and -degrading bacteria in soil and tobacco rhizosphere. Environ. Microbiol..

[143] Webb J.S., Thompson L.S., James S., Charlton T., Tolker-Nielsen T., Koch B., Givskov M., Kjelleberg S. (2003). Cell death in *Pseudomonas aeruginosa* biofilm development. J. Bacteriol..

