# Supplementary material for: Reducing the Impacts of Biofouling in RO Membrane Systems through In Situ Low Fluence Irradiation Employing UVC-LEDs
Source: Membranes (Basel). 2020 Dec 11;10(12):415. doi: 10.3390/membranes10120415 (PMC7764532; doi:10.3390/membranes10120415)
Supplement: Supplementary file 1 [file membranes-10-00415-s001.pdf]

membranes

Article

Supplementary Materials: Reducing the Impacts of Biofouling in RO Membrane Systems through in-situ Low Fluence Irradiation Employing UVC-LEDs

Philipp Sperle, Christian Wurzbacher, Jörg E. Drewes * and Bertram Skibinski *

Chair of Urban Water Systems Engineering, Department of Civil, Geo and Environmental Engineering, Technical University of Munich, Am Coulombwall 3, 85748 Garching, Germany; philipp.sperle@tum.de (P.S.); christian@wurzbacher.cc (C.W.)
* Correspondence: jdrewes@tum.de (J.E.D.); b.skibinski@tum.de (B.S.)

Received: 6 November 2020; Accepted: 3 December 2020; Published: date

1. **Local tap water analysis**

Table S1. Summary of the local tab water analysis.

Parameter	Mean	Standard deviation
TOC [mg·L^{-1}]	1.08	0.79
NO_3-N [mg·L^{-1}]	0.31	0.16
PO_4-P [mg·L^{-1}]	-[1]	-[1]
pH [-]	7.62	0.26
Electrical conductivity [µS·cm^{-1}]	572.49	24.64
TDC [1·mL^{-1}]	4.21 x10^4	1.49 x10^4
Ca [mg·L^{-1}]	60.83	3.2
Cl [mg·L^{-1}]	14.13	2.65
F [mg·L^{-1}]	0.137	0.005
Fe [µg·L^{-1}]	26.80	16.2
K [mg·L^{-1}]	0.98	0.01
Cu [µg·L^{-1}]	18.75	2.22
Ma [mg·L^{-1}]	23.75	0.64
Mn [µg·L^{-1}]	-[1]	-[1]
Na [mg·L^{-1}]	31.65	0.84
SO_4 [mg·L^{-1}]	38.38	5.09

[1] Lower than limit of detection

Anion and cation analysis was performed according to Standard Methods [1–5]. NO_3 and PO_4 were analyzed using cuvette tests (LCK339 and LCK349, Hach, Germany). The remaining anions and cations were analyzed using ion chromatography (Metrohm 930 Compact IC Flex and Metrosep A Supp 7 250 mm, Metrohm, Germany) or atomic absorption spectroscopy (240FS AA and 240Z AA, Agilent, USA).

2. Laboratory skid for biofouling experiments

The laboratory skid for biofouling experiments consists of 2 parallel treatment trains, on with and one without UVC-LEDs (Figure S1). As feed, either deionized (DI) or tap water can be used. Tap water is filtered twice, DI water only once, through a 10 µm PE filter (F) to remove any particulate matter. Next, the water is brought to the desired temperature in a feed tank, submerged in a water bath (TW). The level within the feed tank is controlled by a level sensor (L) connected to a magnetic valve (MV). A magnetic stirrer is mixing the feed water (X2) and the temperature is recorded online (T). Feed water is pumped out of the bottle with the two frequency regulated gear pumps (P2). Shortly after the feed bottle, a nutrient or salt solution can be injected using a dosing pump (P1). The pump speed is measured gravimetrically (S1). Following the dosing, a self-made static mixer ensures that nutrient or salt solutions are equally distributed in the flow. After the mixer, the feed flow splits into 2 parallel treatment trains. In immediate vicinity of the MFS, the in- and outlet pressures are measured (P). Upstream of the MFS, the UV-LED reactor can be attached to one of the trains (within 10 cm distance of the membrane). A "UV-reactor dummy", simply a silica glass pipe with the same dimensions as used in the UV reactor, can be attached to the other train to ensure the same hydraulic retention times in the system. Below the membranes, permeate is collected in a beaker. The flux through the membranes is measured gravimetrically (S2) and recorded online. When the beaker is filled, it is emptied by a suction pump (P3). During the emptying process, the conductivity is recorded (EC2). The MFS, pressure sensors, scales and LED reactors are placed in a temperature-controlled cabinet (TC). Following the MFS, the flow of the concentrate stream is measured using magnetic-inductive flow meters (FM1 and FM2), after which the pressure of the system is regulated by automated needle valves (NV1 and NV2). Using a PI control, the computer aided control system is regulating the flow (measured in the flowmeters) by changing the frequencies of the gear pumps. Finally, after the needle valves, the conductivity of the concentrate is measured (EC1). Data is saved in 5 min intervals. Manufacturer of the parts are summarized in

Table S2.

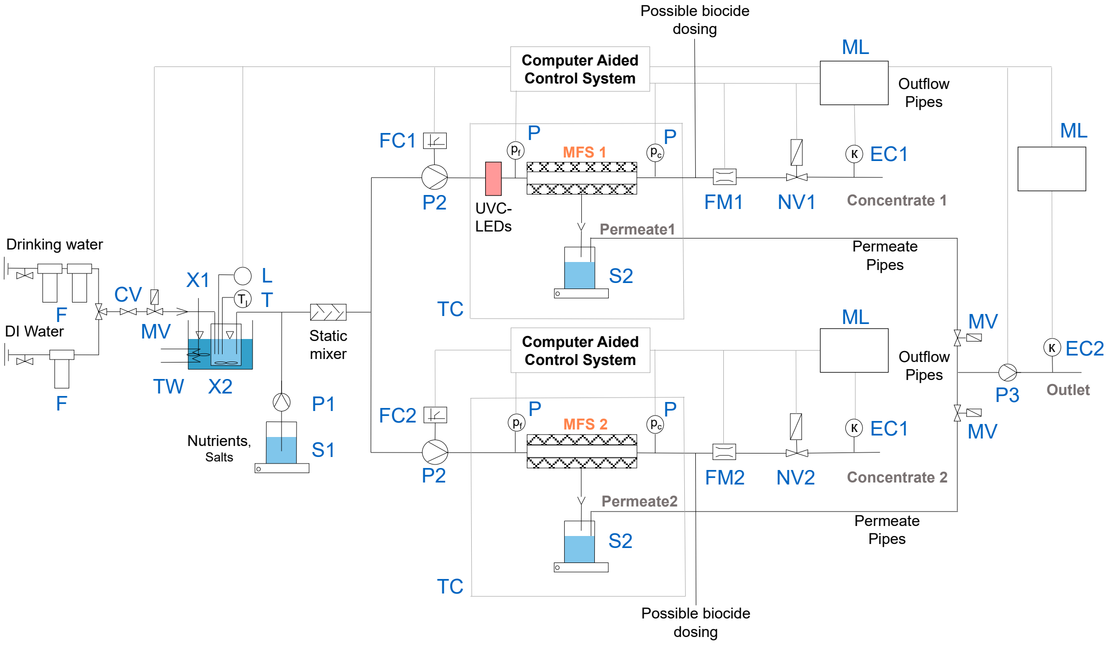

Figure S1. Schematic drawing of the laboratory scale skid for biofouling experiments.

Table S2: Summary of the used material in the laboratory skid for the biofouling experiments.

Symbol	Function	Model	Manufacturer
F	10 μm PE filter	F20 housing and 10 PE filter	MTS & APIC Filter GmbH & Co.KG (Germany)
CV	Control valve	5A111G0414PV	EM-Technik GmbH (Germany)
MV	Magnetic valve	8208066.8050.02400	Buschjost GmbH (Germany)
X1	Stirrer	RZR 2000/D	Heidolph GmbH & Co. KG (Germany)
X2	Magnetic stirrer	17995	Reichelt Chemietechnik GmbH + Co. (Germany)
TW	Water bath and recirculation cooler	UWK 140	Gebrüder HAAKE GmbH (Germany)
L	Level meter	CPA03A2P550A	Schmidt Mess- und Regeltechnik (Germany)
T	Temperature sensor	Pt100 (Biostat)	Sartorius AG (Germany)
P1	Peristaltic pump	DULCO®flex	ProMinent GmbH
S1	Scale	572-49	KERN & SOHN GmbH (Germany)
P2	Gear pump	DGS.38PPPV2NN00000	Tuthill Alsip (United States)
FC1	Frequency converter	FU-E2/370W/IP65	GATHER Industrie GmbH (Germany)
FC2	Frequency converter	FUS 150/EV/IP65	PETER electronic GmbH & Co. KG, (Germany)
TC	Temperature cabinet	Innova 4230	New Brunswick Scientific (United States)
P	Pressure sensor	SML-10	ADZ NAGANO GmbH (Germany)
S2	Scale	572-45	KERN & SOHN GmbH (Germany)
FM1	Magnetic-inductive flow meter	COPA-XM DM23	ABB Automation Products GmbH (Germany)
FM2	Magnetic-inductive flow meter	Proline Promag 10 HART DN02	Endress+Hauser Messtechnik GmbH+Co.KG (Germany)
NV1	Motorized needle valve	Motor: MCL-000AI<br>Valve: SS-4MG2	Motor: Hanbay Inc (United States)<br>Valve: Swagelok (United States)

3. Detailed description of the steps for the accelerated biofouling experiments

The workflow for the biofouling experiments consisted of 4 basic steps:

1. **Cleaning and sterilization:** First, the system (without membrane) was flushed with 0.1% NaOH (Merck, Germany) to remove organic matter [6]. NaOH was recycled for 10 min and afterward soaked for > 12 h, followed by DI water flushing. Next, the disassembled MFS was cleaned using DI water and soap (pure, Baktolin, Germany). The pipe system until the flowmeters was disassembled and autoclaved at 121 °C for 20 min. Membranes were stored at 4 °C in 1% $NaHSO_3$ (Acros Organics, Belgium) till usage [6]. Parts not suitable for autoclaving, including feed and permeate spacer, membrane, MFS, sealing, silica glass pipe, magnetic valve, 10 μm cartridge filters, pressure, temperature and level sensors, were flushed with MilliQ and soaked in 0.25% H_2O_2 solution (Merck, Germany) for at least 24 h to achieve sufficient disinfection (analogous to [6–10]).

2. **Reassembling of the skid:** After the cleaning and sterilization, the skid was reassembled under sterile conditions.
3. **Compaction:** To ensure steady-state conditions for every experiment a compaction of 16 h was performed. Therefore, as feed, DI water was used and a sterile NaCl (Appli Chem, Germany) solution was dosed, resulting in a final concentration of 5.25 mmol·L^{-1} NaCl. The water flow in both lines was set to 4.25 L·h^{-1} and within the first 2 h, a pressure of 8 bar was maintained. The remaining 14 h were used to set a flux of 20 L·m^{-2}·h^{-1} (LMH). The temperature of the feed water was maintained at 15 °C.
4. **Accelerated biofouling phase:** For starting the biofouling experiment, the feed was switched from DI to tap water. UV-LEDs were turned on and nutrients instead of a salt solution were added. Nutrients were sterilized and the pH was set to 10.5 to avoid microbial growth [11]. Nutrient dosing speed was set to reach the aimed concentration of 1,000:200:100 µg·L^{-1}of C:N:P [12]. Roughly every 3 days NaOCl (Merck, Germany) in a 3% solution was dosed for 5 min behind the MFS to avoid clogging of the needle valve. The NaOCl concentration in the concentrate stream was roughly 500 to 1,500 ppm. Throughout the experiment, the feed pressure was kept constant. When a feed channel pressure drop (FCPD) of 0.8 bar was reached, the experiments were terminated.

4. Biofilm extraction

For biofilm extraction, first, the fouled membrane and feed spacer were cut, into approx. 1 cm^2 pieces and placed in a falcon tube containing 30 mL of 0.1 M NaCl. Next, the membrane and spacers were vortexed for 1 h similar as described Matar et al. [13]. After vortexing, samples for ATP, total direct cell count (TDC) and microbial diversity analysis using 16S rRNA sequencing were taken. ATP and TDC analysis were performed within 5 h after the experiment stopped. 16S rRNA samples were frozen at –80 °C till analysis. To separate the EPS from the cells, ultrasonic treatment for 2 min, using 20 W at 20 kHz and the GM 70 HD (Bandelin electronics, Germany) with the UW 70 probe (Bandelin electronics, Germany) and an immersed area of 0.28 cm^2, was applied. Those settings were adapted from Han et al. [14] and kept rather conservative to ensure as low cell lysis as possible. After the ultrasonic treatment, the remaining membrane and spacer pieces were disposed. At this point samples for TOC and fluorescence measurements were taken. The final step consists of a centrifugation at 12,000 rcf and 4 °C for 20 min to separate the cells and the EPS [13]. After cell separation, the EPS was analyzed for its TOC, protein and polysaccharide content. Within 24 h, fluorescence spectroscopy was performed of both, the total biofilm and EPS sample. Samples for protein and polysaccharides analysis were stored at -20 °C. Every analysis except for 16S rRNA amplicon sequencing and fluorescence spectroscopy was performed in triplicates.

5. Aqualog settings

Integration time for fluorescence spectroscopy using the Aqualog (HORIBA Jobin Yvon, Germany) was to 1 s and the CCD gain set to medium. The samples were excited in a 3 nm step from 230 to 599 nm, while recording emission from 211 to 621 nm, each 4 nm. The fluorescence signal was normalized to a daily measure Raman peak (using MilliQ) and corrected for inner filter effects using the HORIBA Scientific and Aqualog V 3.6 software. Besides, a blank was subtracted to account for Raman scattering and Rayleigh scattering areas were set to 0.

6. Actinometry

During the KI/KIO_3 actinometry in a photochemical process I_3^- is formed [15,16]:

$$8KI + KIO_3 + 3H_2O + h\nu \rightarrow 3I_3^- + 6OH^- + 9K^+, \quad (1)$$

the concentration of the produced I_3^- can now be calculated, by measuring the absorbance at 352 nm and dividing this value through the molar absorption coefficient of 27 600 M^{-1}·cm^{-1} [16]. Combining the kinetic of the I_3^- formation over several irradiation times and the wavelength

depending quantum yield, the irradiance of the LED can be calculated over the whole spectrum based on the following equation [17]:

$$I\,[\frac{mW}{cm^2}] = \frac{[I_3^-]\cdot V\cdot h\nu\,\cdot\,N_A}{S\,\cdot t\cdot \Phi}, \tag{2}$$

with $[I_3^-]$ being the concentration of I_3^- ($mol\cdot L^{-1}$), V the solution volume (mL), N the quantity of photons absorbed by the solution (Einstein), Φ the quantum yield of I_3^- ($mol\cdot Einstein^{-1}$), I the UV irradiance/fluence rate ($mW\cdot cm^{-2}$), S the irradiated surface area, t is the irradiation time (s), h is the Planck constant (6.626×10^{-34} J·s), ν the frequency of the wave (s^{-1}) and N_A the Avogadro constant (6.022×10^{23}). As Φ is wavelength dependent, a linear interpolation was used over the whole spectrum as described by [17,18]. $[I_3^-]$/t was estimated as the slope of the several flow steps with hydraulic retention times in the reactor between 0.7 to 6 s. This was seen more appropriate than using a single step as the fitted regression line showed an offset for t=0 (Figure S2). The skid used for actinometry experiments is shown in Figure S3, the parts are described in

Table **S3**.

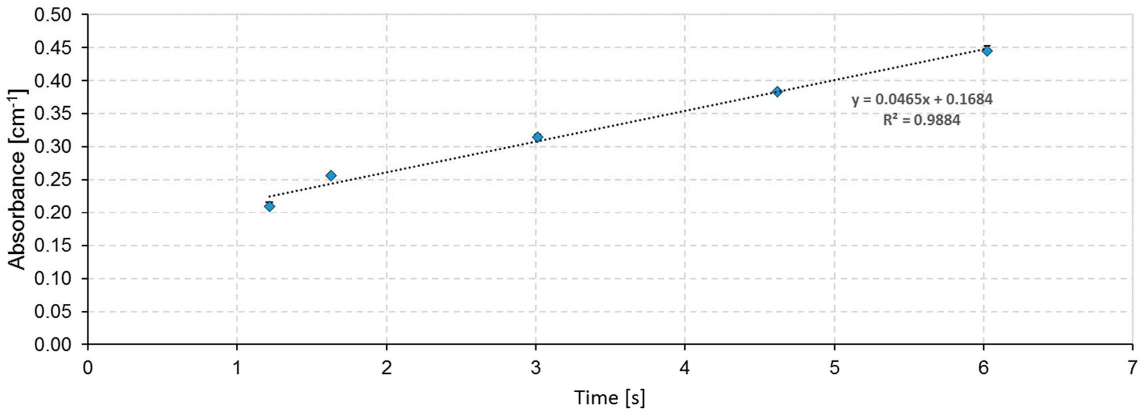

Figure S2. Change of absorbance at 352 nm over hydraulic retention time

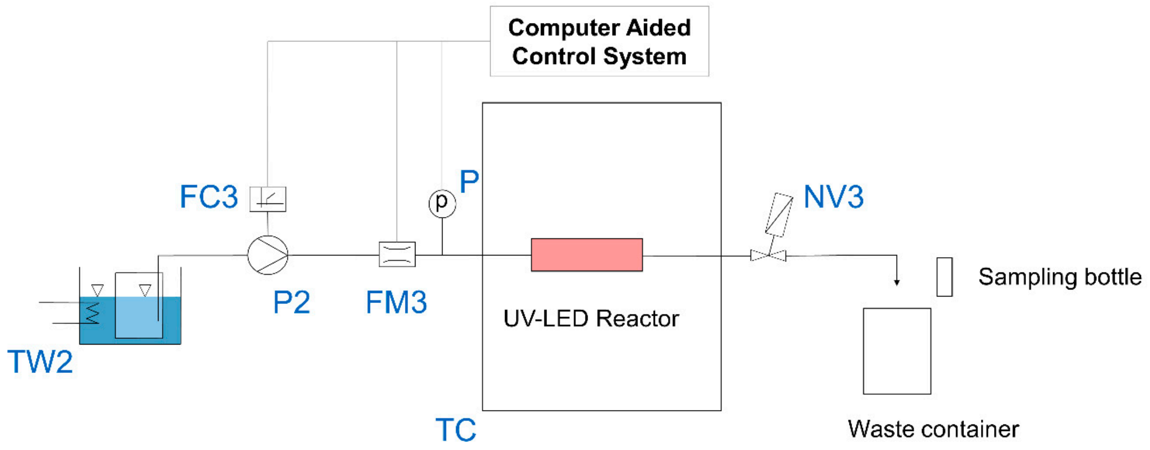

Figure S3. Laboratory skid for actinometry experiments

Table S3. Summary of the used material in the laboratory skid for the actinometry experiments

Symbol	Function	Model	Manufacturer
TW2	Water bath		
P2	Gear pump	DGS.38PPPV2NN00000	Tuthill Alsip (United States)
FC2	Frequency converter	FUS 037/E2	PETER electronic GmbH & Co. KG, (Germany)
FM3	Magnetic-inductive flow meter	MIK-6FC08AC34P	KOBOLD Messring GmbH (Germany
P	Pressure sensor	SML-10	ADZ NAGANO GmbH (Germany)
TC	Temperature cabinet	Innova 4230	New Brunswick Scientific (United States)
NV3	Needle valve	SS-4MG2	Swagelok (United States)

7. Calculation of feed channel pressure drop (FCPD), permeability und hydraulic resistance

Outlier analysis for pressure data and calculated permeability (e.g. caused by an emptying of the beaker collecting the permeate) was done based on the interquartile range [19] (p. 14) of a 3 h interval. FCPD and permeability were calculated as moving average for 1.5 h intervals and at least 5 elements:

$$\text{FCPD [bar]} = \frac{\sum_{i=t-0.75}^{t+0.75} p_{in,i}}{n_{p_{in}}} - \frac{\sum_{i=t-0.75}^{t+0.75} p_{out,i}}{n_{p_{out}}}, \tag{3}$$

with t being the time point in h, p_{in} and p_{out} the in- and outlet pressure and n the number of data points in the interval,

$$\text{Permeability} \left[\frac{\text{LMH}}{\text{bar}}\right] = \frac{\Delta W}{\Delta t\, A} / \left(\frac{\sum_{i=t-0.75}^{t+0.75} p_{in,i}}{2n_{p_{in}}} + \frac{\sum_{i=t-0.75}^{t+0.75} p_{out,i}}{2n_{p_{out}}}\right), \tag{4}$$

With ΔW being the difference in weight [g] measured in the beaker collecting the permeate within the time interval Δt [h] (difference of the first and last value in the 1.5 h interval) and A the membrane area [m^2]. Within calculating the 95% confidence intervals, propagation of uncertainty was considered. The relative permeability is the permeability, normalized to the maximum value found in the first 5 days of the experiment. This value is also used to calculate the hydraulic resistance of the membrane. In general, the total hydraulic resistance can be calculated, adopted from Dreszer et al. [12]:

$$\text{R} \left[\frac{1}{\text{m}}\right] = \frac{1}{\eta\ Permeability}, \tag{5}$$

With R being the total resistance [m^{-1}] and η the dynamic viscosity of water [Pa s] at 15 °C. Further, according to Dreszer et al. [12], the biofilm/fouling layer resistances can be calculated

$$\text{R}_{biofilm} \left[\frac{1}{\text{m}}\right] = R_{total} - R_{membrane}. \tag{5}$$

8. PARAFAC modeling

A three component PARAFAC model was built using 24 samples (Figure S4). For each biofilm sample, an analysis for the EPS and the filtered sample was done. The model was built by first setting areas of Raman and Rayleigh scattering to missing. As a high noise in the UV region was monitored, excitation < 250 and emission < 299 nm was removed. Besides the higher wavelength part for emission > 550 nm was excluded. The model was build out of 10 randomly initialized models using a non-negativity constrain with a convergence of 10-8. For modeling 3 outliers needed to be removed and each EEM was normalized to total intensity.

The PARAFAC models was validated by the variance explained, core consistency and split half analysis. On the one hand, the model showed an explained variance of 98.8% and a core consistency of 86.0%. On the other hand, some limitations for the split-half analysis need to be mentioned. To reduce inner filter effects caused by the high absorbance of the samples, they were diluted 1:20. Whereas in the EEMs pronounced peaks in the protein regions are visible, the signal in the humic substance region was low and prone to measurement noise. This noise can be found in the spectra of the components as well. This noise and the low sample number is subjected to cause a not stable validation with the split-half analysis in random split mode. Anyway, in a randomized $S_4C_6T_3$ split, two of the three comparisons could be validated with a Tucker correlation coefficient of > 95% [20]. The other comparison showed a similarity of 94.6, 89.5 and 89.9% for the three components.

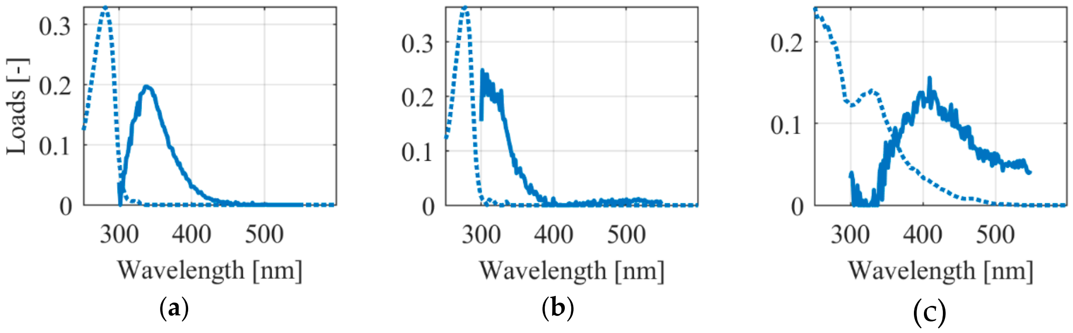

Figure S4. Excitation (dashed line) and emission spectrum (solid line) of the three parallel factors (PARAFAC) model components: (a) Component 1 (C1); (b) Component 2 (C2); (c) Component 3.

9. Distance-based redundancy analysis

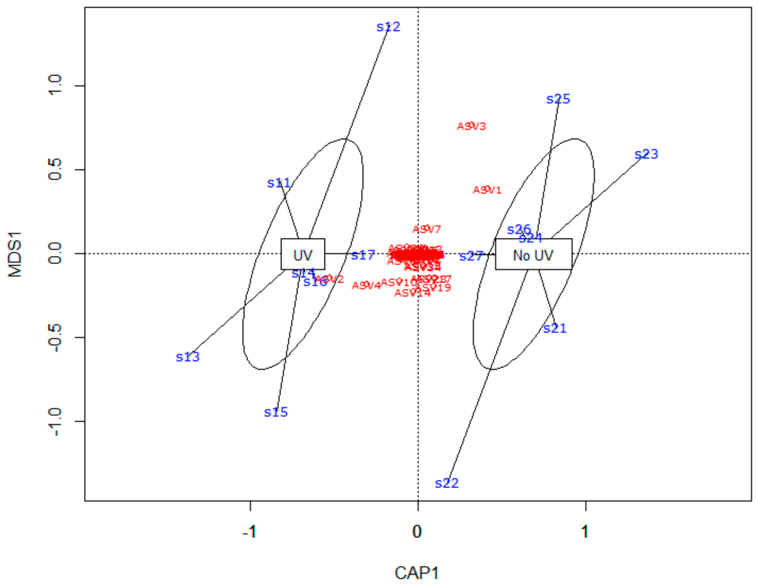

Figure S5. Plot of the distance based redundancy analysis using Bray-Curtis dissimilarity, differentiating for the treatment condition in each experiment. The first number in the sample name represents the treatment condition (2 = untreated, 1 = treated), whereas the second number represents the experimental run.

References

1. *DIN EN ISO 10304-1:2009-07, Wasserbeschaffenheit_- Bestimmung von gelösten Anionen mittels Flüssigkeits-Ionenchromatographie_- Teil_1: Bestimmung von Bromid, Chlorid, Fluorid, Nitrat, Nitrit, Phosphat und Sulfat (ISO_10304-1:2007); Deutsche Fassung EN_ISO_10304-1:2009;* Beuth Verlag GmbH: Berlin.
2. *DIN EN ISO 7980:2000-07, Wasserbeschaffenheit_- Bestimmung von Calcium und Magnesium_- Verfahren mittels Atomabsorptionsspektrometrie (ISO_7980:1986); Deutsche Fassung EN_ISO_7980:2000;* Beuth Verlag GmbH: Berlin.
3. *DIN 38406-6:1998-07, Deutsche Einheitsverfahren zur Wasser-, Abwasser- und Schlammuntersuchung_- Kationen (Gruppe_E)_- Teil_6: Bestimmung von Blei mittels Atomabsorptionsspektrometrie (AAS) (E_6);* Beuth Verlag GmbH: Berlin.
4. *DIN 38405-9:2011-09, Deutsche Einheitsverfahren zur Wasser-, Abwasser- und Schlammuntersuchung_- Anionen (Gruppe_D)_- Teil_9: Photometrische Bestimmung von Nitrat_(D_9);* Beuth Verlag GmbH: Berlin.
5. *DIN EN ISO 6878:2004-09, Wasserbeschaffenheit_- Bestimmung von Phosphor_- Photometrisches Verfahren mittels Ammoniummolybdat (ISO_6878:2004); Deutsche Fassung EN_ISO_6878:2004;* Beuth Verlag GmbH: Berlin.
6. DuPont. *FilmTec™ Reverse Osmosis Membranes. TechnicalManual* Form No. 45-D01504-en, Rev. 2, 2020. https://www.dupont.com/content/dam/dupont/amer/us/en/water-solutions/public/documents/en/45-D01504-en.pdf (accessed on 3 April 2020).
7. Li, K.; Li, S.; Huang, T.; Dong, C.; Li, J.; Zhao, B.; Zhang, S. Chemical Cleaning of Ultrafiltration Membrane Fouled by Humic Substances: Comparison between Hydrogen Peroxide and Sodium Hypochlorite. *Int. J. Environ. Res. Public Health* **2019**, *16*, doi:10.3390/ijerph16142568.
8. Ling, R.; Yu, L.; Pham, T.P.T.; Shao, J.; Chen, J.P.; Reinhard, M. The tolerance of a thin-film composite polyamide reverse osmosis membrane to hydrogen peroxide exposure. *Journal of Membrane Science* **2017**, *524*, 529–536, doi:10.1016/j.memsci.2016.11.041.
9. Kucera, J. Biofouling of Polyamide Membranes: Fouling Mechanisms, Current Mitigation and Cleaning Strategies, and Future Prospects. *Membranes (Basel)* **2019**, *9*, doi:10.3390/membranes9090111.
10. Dow. *FILMTEC™ Reverse Osmosis Membranes. Technical Manual* Form No. 609-00071-0416, n.d.

11. Vrouwenvelder, J.S.; Kruithof, J.; van Loosdrecht, M. *Biofouling of spiral wound membrane systems;* IWA Publishing: [S.l.], 2009, ISBN 9781843393634.
12. Dreszer, C.; Vrouwenvelder, J.S.; Paulitsch-Fuchs, A.H.; Zwijnenburg, A.; Kruithof, J.C.; Flemming, H.-C. Hydraulic resistance of biofilms. *Journal of Membrane Science* **2013**, *429*, 436–447, doi:10.1016/j.memsci.2012.11.030.
13. Matar, G.; Gonzalez-Gil, G.; Maab, H.; Nunes, S.; Le-Clech, P.; Vrouwenvelder, J.; Saikaly, P.E. Temporal changes in extracellular polymeric substances on hydrophobic and hydrophilic membrane surfaces in a submerged membrane bioreactor. *Water Res.* **2016**, *95*, 27–38, doi:10.1016/j.watres.2016.02.064.
14. Han, X.; Wang, Z.; Zhu, C.; Wu, Z. Effect of ultrasonic power density on extracting loosely bound and tightly bound extracellular polymeric substances. *Desalination* **2013**, *329*, 35–40, doi:10.1016/j.desal.2013.09.002.
15. Rahn, R.O. Potassium Iodide as a Chemical Actinometer for 254 nm Radiation: Use of lodate as an Electron Scavenger. *Photochem. Photobiol.* **1997**, *66*, 450–455, doi:10.1111/j.1751-1097.1997.tb03172.x.
16. Rahn, R.O.; Stefan, M.I.; Bolton, J.R.; Goren, E.; Shaw, P.-S.; Lykke, K.R. Quantum Yield of the Iodide–Iodate Chemical Actinometer: Dependence on Wavelength and Concentration¶. *Pure and Applied Chemistry* **2003**, *78*, 146, doi:10.1562/0031-8655(2003)078<0146:qyotic>2.0.co;2.
17. Zou, X.-Y.; Lin, Y.-L.; Xu, B.; Cao, T.-C.; Tang, Y.-L.; Pan, Y.; Gao, Z.-C.; Gao, N.-Y. Enhanced inactivation of E. coli by pulsed UV-LED irradiation during water disinfection. *Sci. Total Environ.* **2019**, *650*, 210–215, doi:10.1016/j.scitotenv.2018.08.367.
18. Wang, W.-L.; Wu, Q.-Y.; Li, Z.-M.; Lu, Y.; Du, Y.; Wang, T.; Huang, N.; Hu, H.-Y. Light-emitting diodes as an emerging UV source for UV/chlorine oxidation: Carbamazepine degradation and toxicity changes. *Chemical Engineering Journal* **2017**, *310*, 148–156, doi:10.1016/j.cej.2016.10.097.
19. Ranga Suri, N.N.R.; Murty M, N.; Athithan, G. Outlier Detection. In *Outlier Detection: Techniques and Applications*; Ranga Suri, N.N.R., Murty M, N., Athithan, G., Eds.; Springer International Publishing: Cham, 2019; pp 13–27, ISBN 978-3-030-05125-9.
20. Murphy, K.R.; Stedmon, C.A.; Graeber, D.; Bro, R. Fluorescence spectroscopy and multi-way techniques. PARAFAC. *Anal. Methods* **2013**, *5*, 6557, doi:10.1039/C3AY41160E.

Publisher's Note: MDPI stays neutral with regard to jurisdictional claims in published maps and institutional affiliations.

© 2020 by the authors. Licensee MDPI, Basel, Switzerland. This article is an open access article distributed under the terms and conditions of the Creative Commons Attribution (CC BY) license (http://creativecommons.org/licenses/by/4.0/).
